# The Antibiotic Negamycin Crosses the Bacterial Cytoplasmic Membrane by Multiple Routes

**DOI:** 10.1128/AAC.00986-20

**Published:** 2021-03-18

**Authors:** Daniel Hörömpöli, Catherine Ciglia, Karl-Heinz Glüsenkamp, Lars Ole Haustedt, Hildegard Falkenstein-Paul, Gerd Bendas, Anne Berscheid, Heike Brötz-Oesterhelt

**Affiliations:** a Interfaculty Institute of Microbiology and Infection Medicine, Department of Microbial Bioactive Compounds, University of Tuebingen, Tuebingen, Germany; b German Center of Infection Research (DZIF), Partner Site Tuebingen, Tuebingen, Germany; c Institute of Pharmaceutical Biology, University of Duesseldorf, Duesseldorf, Germany; d Squarix GmbH, Marl, Germany; e AnalytiCon Discovery GmbH, Potsdam, Germany; f Pharmaceutical Institute, Department of Pharmaceutical & Cell Biological Chemistry, University of Bonn, Bonn, Germany; g Cluster of Excellence 2124: Controlling Microbes to Fight Infection, Tuebingen, Germany

**Keywords:** Dpp, DtpD, *Escherichia coli*, antibiotic, calcium, membrane, natural product, negamycin, peptide transporters, uptake

## Abstract

Negamycin is a natural pseudodipeptide antibiotic with promising activity against Gram-negative and Gram-positive bacteria, including *Enterobacteriaceae*, Pseudomonas aeruginosa, and Staphylococcus aureus, and good efficacy in infection models. It binds to ribosomes with a novel binding mode, stimulating miscoding and inhibiting ribosome translocation. We were particularly interested in studying how the small, positively charged natural product reaches its cytoplasmic target in Escherichia coli. Negamycin crosses the cytoplasmic membrane by multiple routes depending on environmental conditions. In a peptide-free medium, negamycin uses endogenous peptide transporters for active translocation, preferentially the dipeptide permease Dpp. However, in the absence of functional Dpp or in the presence of outcompeting nutrient peptides, negamycin can still enter the cytoplasm. We observed a contribution of the DppA homologs SapA and OppA, as well as of the proton-dependent oligopeptide transporter DtpD. Calcium strongly improves the activity of negamycin against both Gram-negative and Gram-positive bacteria, especially at concentrations around 2.5 mM, reflecting human blood levels. Calcium forms a complex with negamycin and facilitates its interaction with negatively charged phospholipids in bacterial membranes. Moreover, decreased activity at acidic pH and under anaerobic conditions points to a role of the membrane potential in negamycin uptake. Accordingly, improved activity at alkaline pH could be linked to increased uptake of [^3^H]negamycin. The diversity of options for membrane translocation is reflected by low resistance rates. The example of negamycin demonstrates that membrane passage of antibiotics can be multifaceted and that for cytoplasmic anti-Gram-negative drugs, understanding of permeation and target interaction are equally important.

## Abstract (precis)

Negamycin is a natural pseudodipeptide antibiotic with promising activity against Gram-negative and Gram-positive bacteria, including *Enterobacteriaceae*, Pseudomonas aeruginosa, and Staphylococcus aureus, and good efficacy in infection models. It binds to ribosomes with a novel binding mode, stimulating miscoding and inhibiting ribosome translocation.

## INTRODUCTION

Bacterial infections and the increase in antibiotic resistance are among the main health issues of today (1). Discovering and developing new antibiotics against Gram-negative bacteria is particularly challenging. This difficulty is not based on a lack of suitable targets but caused by the strict penetration prerequisites of the Gram-negative cell envelope. In recent years it has become increasingly clear that the failure in identifying new antibiotics with whole-cell activity against Gram-negatives is related to the incomplete understanding of the mechanisms underlying permeation of bacterial membranes (2). An antibiotic needs special characteristics to translocate through the Gram-negative cell envelope, as the outer membrane and cytoplasmic membrane have orthogonal penetration requirements (3–6).

**FIG 1:**
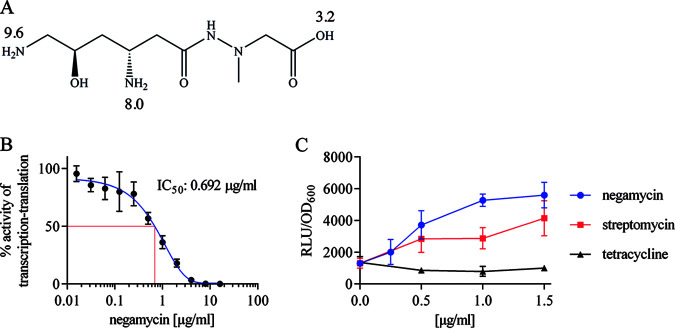
Negamycin structure and mode of action. (A) Structure of negamycin with pKa values (8). (B) Effect of negamycin on coupled *in vitro* transcription-translation using an E. coli S30 extract and plasmid-based Photinus pyralis luciferase as reporter. Error bars showing standard deviation (SD) of five independent experiments. (C) Effect of negamycin, streptomycin (positive control), or tetracycline (negative control) in a whole-cell miscoding assay demonstrating the readthrough of a stop codon within the luciferase gene. Error bars indicating SD of two independent experiments. RLU, relative luminescence units.

To learn from nature, we studied the uptake of the natural product antibiotic negamycin across the cytoplasmic membrane of Escherichia coli. Negamycin is a pseudopeptide with hydroxy-β-lysine as the central amino acid (Fig. 1A). It is a small, hydrophilic compound with a molecular weight of 248.3 g/mol and a positive net charge at neutral pH, discovered already in 1970 in culture filtrates of strains closely related to Streptomyces purpeofuscus (7). In animal models, negamycin cured systemic infections with E. coli, Klebsiella pneumoniae, Salmonella enterica serotype Typhi, Pseudomonas aeruginosa, and Staphylococcus aureus and demonstrated low acute toxicity (7, 8). Interestingly, it is more potent against Gram-negative than against Gram-positive bacteria. Due to its high polarity, it showed low oral bioavailability (6% in rats), low plasma protein binding (10%), and low hepatic clearance, and it was excreted almost entirely via the kidneys in an unmodified form (8).

Negamycin inhibits translation. Early studies reported an inhibition of ribosome translocation, stabilization of polysomes, disturbance of the termination process, and miscoding (9–13). In crystal structures, the compound was found bound to several sites of the small and large ribosomal subunits (14–16). Resistance mutations in a strain carrying only one rRNA allele mapped the primary site of antibiotic action to helix 34 of the 16S rRNA, a position that overlaps with the tetracycline binding site. However, in contrast to tetracycline, negamycin also establishes contacts with the aminoacyl-tRNA and increases the residency time of noncognate tRNAs (14). In accordance with this miscoding activity, negamycin is bactericidal (10). Negamycin triggers miscoding at the eukaryotic ribosome as well and cured Duchenne muscular dystrophy in mice, which carried a nonsense mutation in the dystrophin gene (17, 18).

In an attempt to improve the efficacy of negamycin, several derivatization campaigns were conducted by companies and academic groups, which almost exclusively resulted in a loss of activity (19–21). Only a single recently reported derivative, N6-(3-aminopropyl) negamycin, showed 4-fold improved antibacterial activity (22). Notably, among the derivatives generated over the years, several were active in ribosomal extracts but failed in whole-cell MIC assays, suggesting uptake issues (23). This observation and the fact that negamycin activity had displayed strong media dependency (7) stimulated our interest in studying the uptake process of the agent across the cell envelope. For optimization of negamycin, a thorough understanding of the uptake mechanism seems equally important as detailed insight into the target interaction.

When we started our investigations, we were aware of a poster presented by Versicor Inc. at the Interscience Conference on Antimicrobial Agents and Chemotherapy (ICAAC) already in 2002 (24) demonstrating that E. coli mutants with a defective dipeptide permease Dpp or deficient in components of the electron transport chain show low-level resistance to negamycin. While our work was in progress, a publication by AstraZeneca confirmed these findings and showed that Dpp plays a minor role in negamycin uptake during treatment of an E. coli mouse thigh infection (25).

In our studies, with a mechanistic focus in mind, we compared growth media of entirely different composition, on the one hand, M9 minimal medium rich in salt and glucose but free of peptides, versus on the other hand, 0.5% polypeptone (PP) in water containing a nondefined mixture of peptides but no externally added sugars, salts, or buffer. Here, we report on the passage of negamycin across the cytoplasmic membrane of E. coli and demonstrate that more than one route can be used, with their respective contributions determined by the environment. The complex uptake process of negamycin shows that more than one entry mechanism should be considered when studying natural product passage into bacterial cells. Evolution can bestow natural products with a variety of interactions facilitating entry, which makes them valuable models for studying antibiotic uptake.

## RESULTS

### Media conditions significantly affect negamycin activity.

Negamycin used in this study was of synthetic origin and inhibited translation in an E. coli cell-free system with a half-maximal inhibitory concentration (IC_50_) of 2.8 μM (0.69 μg/ml, Fig. 1B), in accordance with previously published values (20, 22, 26). The compound also induced stop codon readthrough in an E. coli whole-cell miscoding assay (Fig. 1C). The antibacterial activity of negamycin against E. coli varied substantially in growth media of different compositions. In rich media, such as Mueller-Hinton broth (MHB) and lysogeny broth (LB), MICs were greater than or equal to 64 μg/ml (Table 1). Markedly stronger antibacterial activity was detected in M9 or PP, corresponding to MICs of 4 μg/ml and 8 μg/ml for E. coli strain BW25113, respectively. Pseudomonas aeruginosa strain PAO1 was also inhibited, although at higher concentrations (32 to 64 μg/ml), while the Gram-positive bacteria tested (i.e., Staphylococcus aureus strain ATCC 29213, Bacillus subtilis strain 168 *trpC2*) were not inhibited up to 64 μg/ml under these conditions (Table 1), demonstrating that negamycin is stronger against Gram-negatives. As comparators, we used the antibiotics ciprofloxacin, tetracycline, and gentamicin for MIC determinations in the same set of media. We did not detect any large differences in activity between the four media for these reference antibiotics, with the exception of the decreased activity of gentamicin in media containing a larger amount of salt (i.e., LB and M9), which was observed across different species (Table 1). The activity of negamycin in media of orthogonal composition was a first indication of the use of multiple entry routes into the bacterial cytoplasm. To dissect these uptake mechanisms, we chose M9 as well as PP for all further experiments.

**TABLE 1:** Antimicrobial activity of negamycin and the reference antibiotics ciprofloxacin, tetracycline, and gentamicin in different growth media

	MIC (μg/ml)															
	Negamycin				Ciprofloxacin				Tetracycline				Gentamicin			
	MHB	LB	M9	PP	MHB	LB	M9	PP	MHB	LB	M9	PP	MHB	LB	M9	PP
E. coli BW25113	64	64	4	8	0.016	0.016	0.008	0.008	1	2	1−2	2	0.5	4	0.25	0.06
E. coli ATCC 25922	>64	>64	2^*a*^	16	0.008	0.008	0.008^*a*^	0.004	1	1	0.5^*a*^	1	1	8	0.5^*a*^	0.125
P. aeruginosa PAO1	>64	>64	64	32	0.06	0.06	0.06	0.06	16	16	16	8	0.25	2	2	0.25
S. aureus ATCC 29213	>64	>64	ng	>64	0.25	0.25	ng	0.125	0.5	0.5	ng	1	0.25	4	ng	0.25
B. subtilis 168 *trpC2*	>64	>64	ng	>64	0.06	0.06	ng	0.06	4	8	ng	8	0.06	0.25	ng	0.03

*^a^*M9 minimal medium supplemented with thiamine (1 mg/liter) for E. coli ATCC 25922. ng, no growth.

### Negamycin crosses the cytoplasmic membrane via different endogenous E. coli peptide transporters.

**FIG 2:**
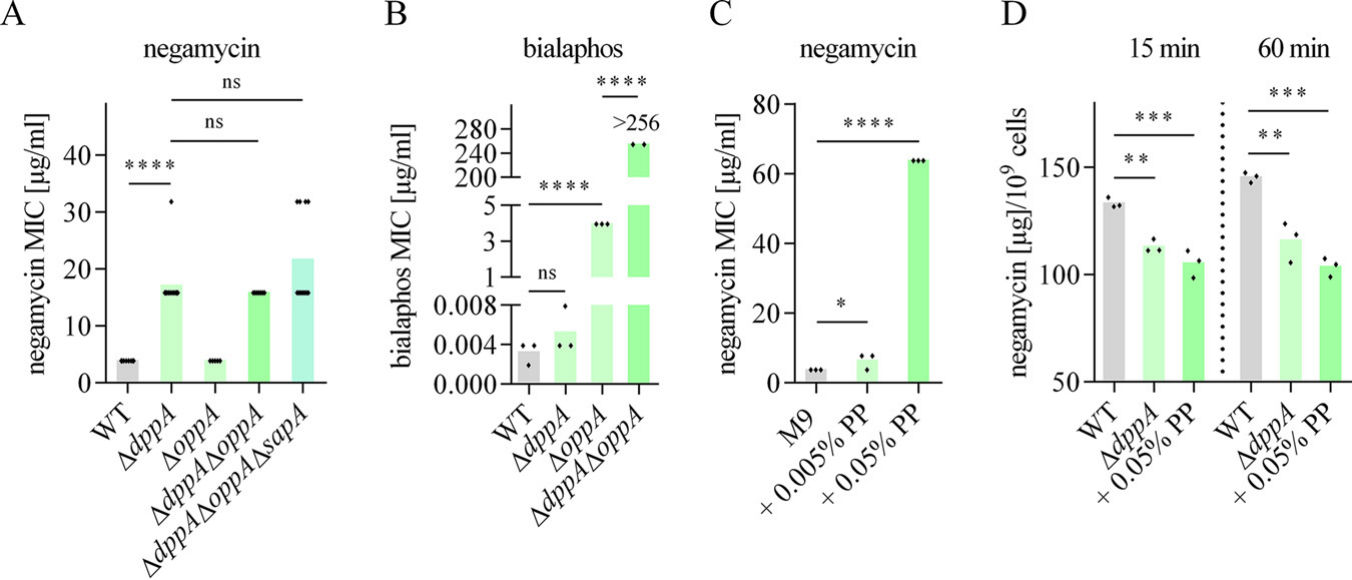
Negamycin uses multiple peptide transporters and competes with peptides for uptake, whereas entry options for bialaphos are limited to Opp and Dpp transporters. (A and B) Impact of single or multiple ABC peptide transporter deletions in the E. coli BW25113 background on negamycin (A) or bialaphos (B) susceptibility in M9 medium. (C) Impact of peptide addition on negamycin MICs in M9. Negamycin susceptibility of E. coli BW25113 in M9 decreases with supplemented polypeptone (PP) in a concentration-dependent manner. (D) Uptake of [^3^H]negamycin into E. coli BW25113 wild type or its isogenic Δ*dppA* mutant in M9. [^3^H]negamycin (specific activity: 0.052 Ci/mmol) was added to the cells at a concentration of 32 μg/ml. One sample of the wild type was supplemented with 0.05% PP concurrently with negamycin addition. Samples were taken after 15 (left) or 60 (right) min of incubation at 37°C with shaking. Negamycin uptake is significantly reduced in M9 with the addition of 0.05% PP or the deletion of the peptide transporter gene *dppA*. Each diamond represents an independent MIC determination (A to C) or [^3^H]negamycin uptake measurement (D). Statistical significance was determined using unpaired Student’s *t* test with Holm-Bonferroni correction. ns, *P* > 0.05; *, *P* ≤ 0.05; **, *P* ≤ 0.01; ***, *P* ≤ 0.001; ****, *P* ≤ 0.0001.

Due to its pseudopeptide-like structure and previous reports on the dipeptide permease Dpp as an entry route into E. coli (24, 25), we investigated whether negamycin is capable of using one or more additional peptide transporters. Aiming at a comprehensive picture, we tested a large variety of E. coli peptide as well as amino acid transporter mutants (Table 2). Dpp mutants elicited the strongest effect. Single knockouts of each of the genes encoding the different subunits of the ABC transporter Dpp displayed a 4-fold increase in the negamycin MIC in M9 medium (Table 2), which is in accordance with previous reports (24, 25). Expressing *dppA* from a plasmid complemented the *dppA* deletion (Fig. S1). To prove that the decreased negamycin susceptibility observed in MIC determinations is directly linked to a reduced antibiotic uptake, we performed experiments with radiolabeled negamycin. Here, we detected a significantly decreased [^3^H]negamycin uptake into the Δ*dppA* mutant compared to its isogenic parent strain, with a 15% smaller amount after 15 min and a 20% smaller amount after 60 min of treatment (Fig. 2D). Since negamycin is still capable of inhibiting the growth of *dpp* knockout mutants, additional uptake routes into the cytoplasm must be available. The knockout of *sapA*, a paralog of *dppA*, led to a 2-fold increase in the negamycin MIC, while the deletion of the other genes encoding the Sap ABC transporter (i.e., permease domains *sapB* and *sapC*, ATPase domains *sapD* and *sapF*) did not reduce negamycin susceptibility (Table 2). No clear effects were detected in single-knockout mutants of the genes encoding the different subunits of the main E. coli oligopeptide transporter Opp (Table 2). SapA and OppA share 36% and 25% amino acid identity with DppA, respectively. The deletion of the genes encoding DppA paralogs like DdpA, GsiB, MppA, NikA, and YgiS did not reduce negamycin susceptibility (Table 2). To detect putative cross talk or compensatory effects due to redundancy of paralogous periplasmic binding proteins of different ABC transporters, double and triple knockout mutants were generated. The double knockouts Δ*dppAΔoppA* and Δ*dppAΔsapA* did not display a higher negamycin resistance than Δ*dppA* alone. The Δ*dppAΔoppAΔsapA* triple deletion mutant showed a marginal, nonsignificant increase in the negamycin MIC compared to the Δ*dppA* single-knockout (Table 2 and Fig. 2A). The herbicide bialaphos (l-alanyl-l-alanyl-phosphinothricin) is a tripeptide that has been described as a substrate of mainly the Opp transporter, but it can also use Dpp as a second route for cell entry (27). When comparing bialaphos to negamycin, it became apparent that bialaphos has limited options for cell entry apart from the two main E. coli peptide ABC transporters Opp and Dpp. Single deletion of *oppA* already increased the MIC dramatically (1,000-fold), and, although a single deletion of *dppA* did not show a significant effect, the MIC rose more than 64,000-fold in a Δ*oppA*Δ*dppA* double mutant (Fig. 2B). In contrast, only a 4-fold increase in negamycin MIC was observed in both the Δ*dppA* single as well as the Δ*dppAΔoppA* double knockout mutants (Fig. 2A), confirming that negamycin has considerably more options of entering the cytoplasm than bialaphos. In addition to the ABC transporters mentioned above, the deletion of one of the four E. coli proton-dependent oligopeptide transporters (POTs), namely, DtpD (YbgH), led to a 2-fold decrease in negamycin susceptibility (Table 2). When we additionally deleted *dtpD* in the Δ*dppA*Δ*oppA*Δ*sapA* mutant background, the resulting quadruple mutant showed a 2-fold negamycin MIC increase compared to that of Δ*dppA* and consequently an 8-fold MIC increase compared to that of the wild type in M9 medium (Table 2). Notably, none of the multiple knockout mutants showed complete negamycin resistance, indicating the presence of further negamycin uptake routes and revealing the highly promiscuous nature of this natural product antibiotic.

**TABLE 2:** Negamycin MICs of various E. coli BW25113 transporter mutants in M9 or 0.5% polypeptone (PP)

	Negamycin MIC (μg/ml)^*d*^	
E. coli strain	M9	PP
BW25113 wild type	4	8
Single transporter deletions^*a*^		
ATP-dependent oligopeptide transporters		
Δ*dppA,* Δ*dppB,* Δ*dppC,* Δ*dppD,* Δ*dppF*	**16**	8
Δ*oppA,* Δ*oppB,* Δ*oppC,* Δ*oppD,* Δ*oppF*	4	8
Δ*sapA*	**8**	**16**
Δ*sapB,* Δ*sapC,* Δ*sapF*	4	8
Δ*sapD*	2	8
Δ*ddpA, ΔgsiB, ΔmppA, ΔnikA, ΔygiS*	4	8
Proton-dependent oligopeptide transporters		
Δ*dtpD* (Δ*ybgH*)^*b*^	**4–8**	**16**
Δ*dtpA* (Δ*tppB*), Δ*dtpB* (Δ*yhiP*), Δ*dtpC* (Δ*yjdL*)	4	8
Amino acid transporters		
Δl*ysP*, Δ*hisP*	4	nd
Δ*cadB*, Δ*hisQ*, Δ*hisJ*, Δ*argT*	4	nd
Δ*hisM*	2–4	nd
Multiple transporter deletions^*c*^		
Δ*dppA*Δ*oppA*	**16**	**8–16**
Δ*dppA*Δ*sapA*	**16**	8
Δ*dppA*Δ*dtpD*	**16**	8
Δ*dppA*Δ*oppA*Δ*sapA*	**16–32**	8
Δ*dppA*Δ*oppA*Δ*sapA*Δ*dtpD*	**32**	8

a Strains originating from the Keio collection (76).

b In cases where genes of previously unknown function were later renamed according to their function, the names in parentheses refer to names employed in the Keio collection.

c Strains generated in the course of this work. nd, not determined.

d Numbers in bold indicate increases in the MIC compared to the parent strain E. coli BW25113.

Generally, the effects of peptide transporter deletions on negamycin susceptibility were significantly more pronounced in M9 than in PP medium (Table 2). Here, the orthogonal composition of our two growth media is relevant. In M9 lacking any peptide ingredient, negamycin has free access to peptide transporters. In contrast, in PP, where peptides represent the sole carbon and nitrogen source, nutrient peptides are highly abundant, and negamycin is under strong competition with them at the transporters. The unaltered negamycin susceptibility of the *dpp* mutants in PP compared to that of the wild type demonstrates that here, negamycin entry is not dominated by passage through Dpp. Accordingly, when peptides (i.e., polypeptone) were added to M9 medium, the negamycin MIC increased in a concentration-dependent manner (Fig. 2C) and to a level surpassing that of the *dppA* knockout, which suggests a role of peptide transporters unrelated to Dpp. Likewise, we observed that significantly less [^3^H]negamycin accumulated in E. coli after addition of 0.05% polypeptone to M9 medium in radioactive uptake experiments (Fig. 2D). The activity of negamycin against multiple peptide transporter knockouts as well as in peptide-containing media indicates that negamycin must enter the cell via at least one additional uptake route.

### Negamycin resistance frequencies reflect the availability of multiple uptake routes.

**FIG 3:**
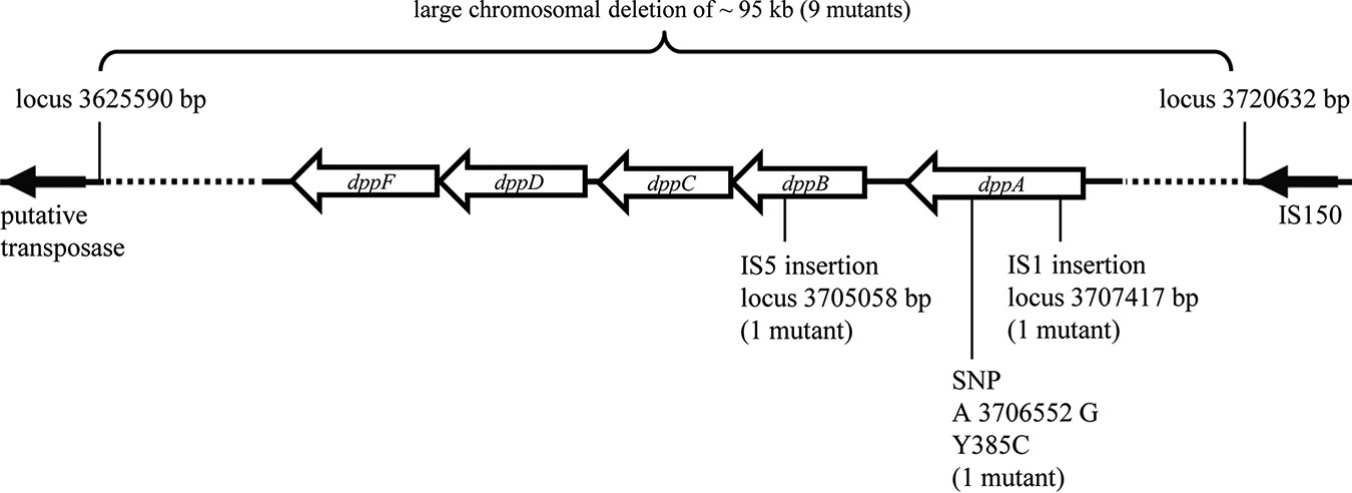
Schematic overview of the *dpp* operon in E. coli and spontaneous mutations induced by negamycin exposure. Indicated deletions, IS element insertions, and a point mutation were detected in spontaneous negamycin resistant mutants isolated from M9 agar plates containing 2× MIC negamycin. Locus numbers based on E. coli K-12 MG1655 (GenBank access no. U00096.3). SNP, single nucleotide polymorphism.

Strong preference for a single uptake route versus the option of multiple entry routes is also reflected by the risk of acquiring high-level resistance. Thus, we next compared the mutation frequencies of negamycin in our two selected growth media and first concentrated on the situation close to the MIC. In M9, the resistance frequency at 2× MIC for negamycin in E. coli BW25113 was 6 × 10^−7^. As *dpp* deletion had shown the strongest impact on negamycin activity in M9 (Table 2), we next analyzed the *dpp* operon of 12 E. coli BW25113 mutants isolated from M9 agar at 2× MIC. No PCR product was obtained for 9 of the 12 selected mutants when using primers flanking the *dpp* operon (dppA-F-2 and dppA-F rev2). Whole-genome sequencing of one of these mutants revealed a large chromosomal deletion of approximately 95 kB, containing the entire *dpp* operon plus large flanking regions (Fig. 3). This large deletion was subsequently also identified in the 8 other mutants that initially had not yielded a PCR product for the *dpp* operon. Interestingly, two mobile genetic elements (putative transposase *yhhI* upstream and IS150 downstream) flank the deleted region, which might have facilitated excision. The deletion had occurred at the start of a REP260 element and fused this part to the inverted repeat left (IRL) of IS150. A similar deletion (approximately 100 bp smaller) had been detected in negamycin resistant mutants in an E. coli W3110 background (25). When we conducted a BLAST search, we found that the same deletion had also occurred in an E. coli K-12 MG1655 strain during exposure to subinhibitory concentrations of the antibacterial phenolic monoterpene carvacrol (sequence ID CP026026.1 [28]). According to the Profiling of E. coli Chromosome database (PEC; shigen.nig.ac.jp/ecoli/pec), no essential genes are found in this 95-kB region of the chromosome (29). With regard to our three remaining mutants isolated from M9 agar under negamycin selection pressure, one mutant harbored a point mutation in *dppA* that led to a Y385C amino acid exchange, while two of the mutants had insertions of mobile genetic elements in the *dpp* operon. Among these, one mutant carried an insertion of the insertion element IS1 in *dppA*, and in the second mutant, IS5 had integrated into *dppB* (Fig. 3). In total, all 12 out of 12 E. coli BW25113 mutants isolated on M9 agar at 2× MIC showed alterations in the *dpp* operon, confirming Dpp as the preferential uptake route for negamycin in peptide-free media. The negamycin MIC of all mutants was increased by 4-fold in M9 (MIC, 16 μg/ml), in agreement with the genetic knockout of *dppA* and other genes of the *dpp* operon. In PP medium, the MIC of all mutants was unchanged.

While the resistance frequency for negamycin was high at 2× MIC in M9, it already strongly decreased at 4× MIC (<7 × 10^−9^). This number reflects our limit of detection and might be even lower. For comparison, for the tripeptide bialaphos, mutation frequencies in M9 were high even at 100× MIC (frequencies of 4 × 10^−6^ at 4× MIC, 4 × 10^−7^ at 10× MIC, and 7 × 10^−7^ at 100× MIC). This result confirms that loss of function of a single transporter already leads to a large decrease in bialaphos activity, i.e., a 1,000× MIC increase in a Δ*oppA* strain (Fig. 2B). On PP agar, negamycin resistance frequencies were similar to those on M9, with a high frequency of 5 × 10^−7^ at 2× MIC and less than 7 × 10^−9^ at 4× MIC. However, in contrast to M9, no alterations in the *dpp* operon were detected in 8 mutants isolated from PP agar, confirming that Dpp does not play a prominent role in negamycin transport in peptide-containing media. Importantly, the low mutation rate at 4× MIC in both very different media indicates that no high-level negamycin resistance can be obtained by mutation of a single transporter, as other uptake routes remain available.

### Negamycin activity is significantly improved by calcium.

**FIG 4:**
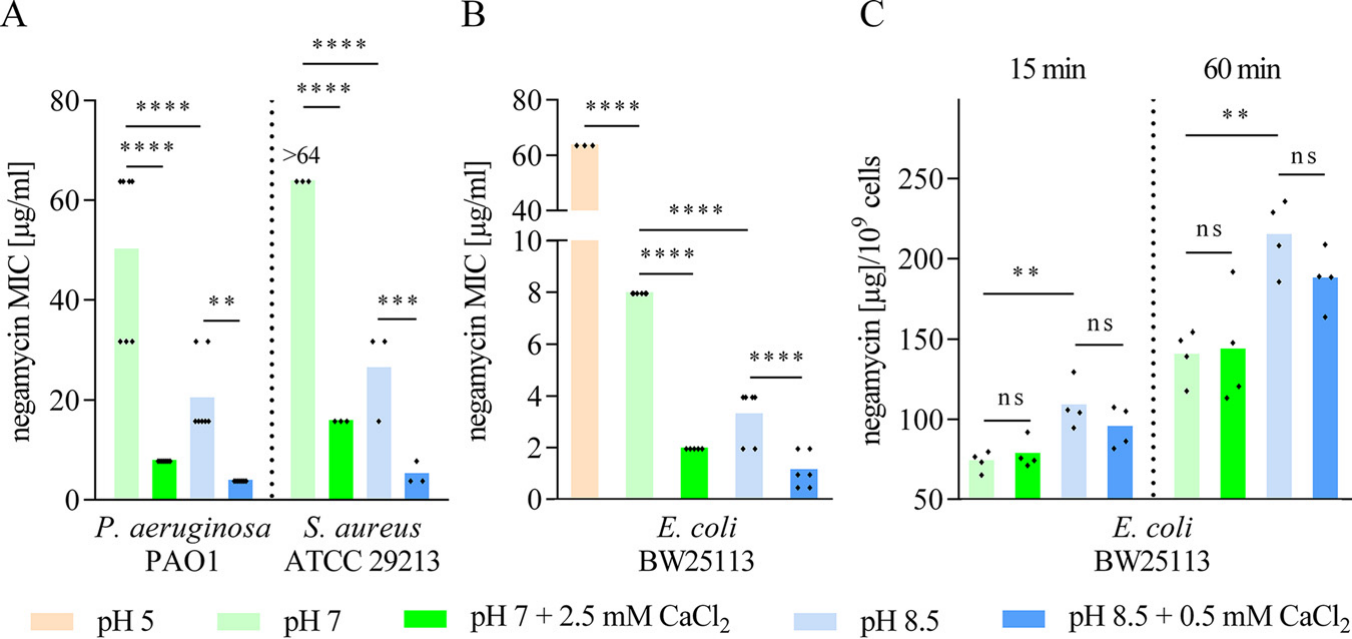
Basic pH and calcium are additive in improving negamycin activity. Negamycin MICs of P. aeruginosa PAO1 and S. aureus ATCC 29213 (A) and of E. coli BW25113 (B) in 0.5% PP medium adjusted to different pH values and in combination with CaCl_2_. (C) Influence of pH and CaCl_2_ on [^3^H]negamycin uptake in E. coli BW25113 in PP. A total amount of 32 μg/ml of [^3^H]negamycin (specific activity: 0.052 Ci/mmol) was added to the cells. CaCl_2_ and [^3^H]negamycin were added to the cultures in parallel. Samples were taken after 15 (left) or 60 (right) min of incubation with [^3^H]negamycin at 37°C with shaking. Alkaline pH increased [^3^H]negamycin uptake, while we could not detect such an effect after the addition of CaCl_2_. Each diamond represents an independent MIC determination (A and B) or [^3^H]negamycin uptake measurement (C). Statistical significance was determined using unpaired Student’s *t* test with Holm-Bonferroni correction. ns, *P* > 0.05; **, *P* ≤ 0.01; ***, *P* ≤ 0.001; ****, *P* ≤ 0.0001.

Next, we investigated the impact of different salt conditions on negamycin activity in PP medium, which does not contain salts *a priori*. The addition of 0.5 mM CaCl_2_ to the medium improved the negamycin MIC in E. coli BW25113 by 2-fold, while a concentration of 2.5 mM was the most effective and led to a 4-fold decrease of the negamycin MIC (Table 3). Calcium also had a beneficial effect on negamycin activity against other species, e.g., P. aeruginosa and S. aureus (Fig. 4A). MICs dropped by 2- to 4-fold when cation-adjusted MH broth (MHBII) compared to standard MHB was used. MHBII contains calcium at a concentration of 20 to 25 μg/ml, corresponding to approximately 0.5 mM. Magnesium, on the other hand, had no significant effect. Other divalent cations (i.e., Mn^2+^, Zn^2+^, and Ni^2+^) could not be tested in the same concentration range as calcium and magnesium due to toxicity for the E. coli cells. Low concentrations of NaCl did not improve negamycin activity, and the addition of 50 mM NaCl was even inhibitory, as were other monovalent cations like K^+^ and NH_4_^+^ (Table 3). On a side note, when reducing the total salt concentration of M9 to one-fourth of its regular content (and leaving the glucose amount unchanged), the negamycin MIC dropped from 4 μg/ml to 1 μg/ml for E. coli. M9 contains monovalent cations (Na^+^, K^+^, NH_4_^+^), the sum of which amounts to approximately 145 mM. Thus, the monovalent cations present in M9 may negatively affect activity in this medium, especially the Dpp unrelated routes. For comparison, the MICs of ciprofloxacin, tetracycline, and gentamicin were also determined in PP in the presence of different salts. Here, CaCl_2_ and MgCl_2_ did not improve activity of these compounds and rather increased their MICs at 50 mM respective salts (Table S1). As observed for negamycin, NaCl decreased the activity of the aminoglycoside gentamicin, while ciprofloxacin and tetracycline MICs remained unaffected (Table S1).

**TABLE 3:** Negamycin activity against E. coli BW25113 in the presence of different concentrations of salts added to 0.5% polypeptone medium

	Negamycin MIC (μg/ml)^*a*^				
Salt concentration	CaCl_2_	MgCl_2_	NaCl	KCl	NH_4_Cl
0 mM	8	8	8	8	8
0.5 mM	**4**	8	8	8	8
2.5 mM	**2**	8	8	8	8
5 mM	**2–4**	8	8	**8–16**	**8–16**
10 mM	**4**	8	8	**8–16**	**16**
50 mM	8	8	**32–64**	**32**	**64**

a Numbers in bold indicate changes in the MIC compared to activity without salts.

In a published ribosome cocrystal structure, negamycin established contacts to the rRNA as well as the tRNA by coordinating a magnesium ion (14). Not to overlook a potential benefit of calcium at the target level, we performed the E. coli *in vitro* translation assay and added 1 to 8 μM calcium to the assay mixture. The IC_50_ of negamycin remained unchanged. E. coli keeps its cytoplasmic free Ca^2+^ concentration low and tightly regulated (steady-state levels of 200 to 300 nM), even when exposed to external Ca^2+^ concentrations in the millimolar range (30), while cytoplasmic magnesium concentrations are much higher (1 to 5 mM free Mg^2+^ [31]). Therefore, it was unlikely that the beneficial effect that external calcium exerted on negamycin activity was related to target binding.

### Calcium enhances binding of negamycin to phospholipid membranes.

**FIG 5:**
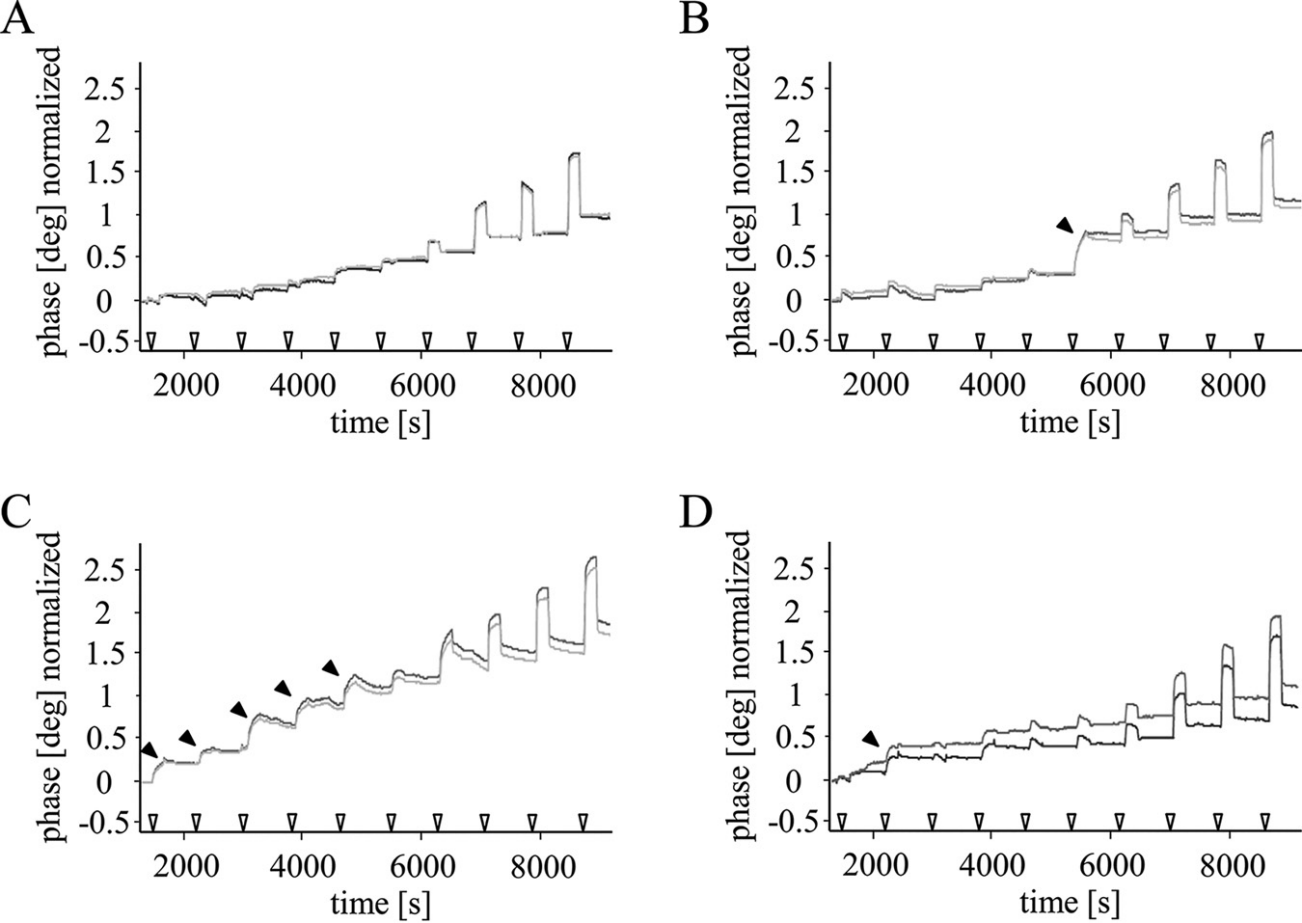
Calcium supports the binding of negamycin to phospholipid membranes. Surface acoustic wave (SAW) sensorgrams comparing the interaction of negamycin with a POPC membrane (A), a POPC/DOPG membrane (B), or a POPC/DOPG membrane in the presence of 2.5 mM CaCl_2_ (C) or with a POPC/DOPG membrane in the presence of 2.5 mM MgCl_2_ (D). The empty triangles at the *x* axis indicate the injection steps of negamycin. The total concentration of negamycin ranges from 7.5 × 10^−8^ M (outermost left triangle) to 1 × 10^−5^ M (outermost right triangle). Filled arrows at the curves indicate binding events. The large phase shifts at high negamycin concentrations which drop back after injection are a consequence of increased viscosity and do not indicate real binding events.

To investigate the interaction of negamycin with a phospholipid membrane, we performed surface acoustic wave (SAW) biosensor measurements. A model membrane bilayer consisting of a lower lipid layer immobilized at the sensor surface and an upper phospholipid layer was exposed to negamycin in a flow chamber, and binding events were recorded. As phospholipids, we compared, on the one hand, 1-palmitoyl-2-oleoyl-phosphatidylcholine (POPC) and, on the other hand, a mixture of 90% POPC (net neutral)/10% 1,2-dioleoyl-phosphatidylglycerol (DOPG, net negative) to provide the negative charge common to bacterial cytoplasmic membranes. Each experiment started with the injection of negamycin at nanomolar concentrations, and further negamycin was added to the system by serial injections up to the micromolar range. SAW experiments showed that negamycin already has a certain binding tendency to an uncharged POPC model membrane indicated by the small phase change starting at the fourth injection (Fig. 5A). Binding of negamycin was substantially increased by adding 10% of DOPG to the POPC membrane, as shown by the earlier onset and the higher degree of binding (Fig. 5B). Furthermore, the presence of 2.5 mM CaCl_2_ strongly improved the interaction of negamycin with the membrane, clearly indicated by the rapid onset of binding already at the lowest negamycin concentration and the larger phase change (Fig. 5C). The cation species appears to be crucial for the membrane-binding affinity, as the addition of 2.5 mM MgCl_2_ had only a marginal effect on the binding process (Fig. 5D). The observation that CaCl_2_ improved negamycin activity not only against Gram-negative but also against Gram-positive bacteria (Fig. 4A) suggests that calcium facilitates negamycin uptake at the cytoplasmic membrane. However, an additional beneficial effect at the outer membrane cannot be excluded at this point.

To rule out secondary antibiotic effects emerging from the interaction of negamycin with phospholipid membranes stimulated by calcium, we determined the membrane potential and permeability in E. coli upon negamycin treatment. The membrane potential was unaffected up to 4× MIC of negamycin over a time course of 180 min in the absence and presence of calcium (Fig. S2). We also could not observe an increased membrane permeability within 60 min of negamycin treatment as monitored by staining with the DNA-binding, cell-impermeant fluorescent dye SYTOX Green (Fig. S3). In contrast, colistin increased membrane permeability within 10 to 20 min of treatment. After 90 to 120 min of negamycin treatment, the SYTOX Green assay signaled a beginning impairment of membrane integrity, an effect probably related to the miscoding activity of this compound (Fig. S3). Importantly, the addition of calcium did not promote the disruption of membrane integrity; on the contrary, it rather seemed to have a stabilizing effect on the cells.

### Negamycin forms a complex with calcium.

**FIG 6:**
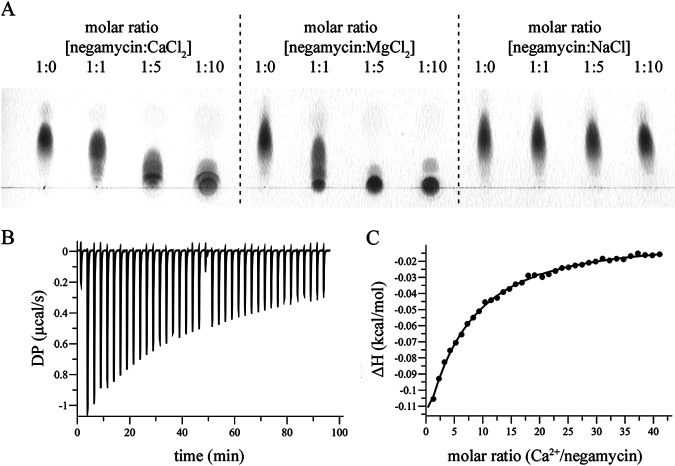
Negamycin forms a complex with calcium. (A) Thin-layer chromatography (TLC) analysis of negamycin with CaCl_2_, MgCl_2_, or NaCl at different molar ratios. (B and C) Isothermal titration calorimetry (ITC) of negamycin titrated with CaCl_2_. (B) Heat differences monitored by differential power (DP) measurements upon two consecutive series of 19 injections of CaCl_2_ after baseline correction and subtraction of control experiments of CaCl_2_ titration into buffer, buffer titration into buffer, and negamycin titration into buffer. (C) Binding enthalpies (ΔH) against the Ca^2+^/negamycin molar ratio. ITC data were fitted to the one-site binding model. Due to the low binding affinity, fitting required to preset stoichiometry manually, which we set to 1:1. With these settings, a K_d_ of 7.98 mM was obtained.

We further tested the capacity of negamycin to interact with calcium directly. Mainly the carboxyl, but also the carbonyl and amine groups within the flexible negamycin molecule, indicate that it may be able to chelate cations. In thin-layer chromatography (TLC), the addition of Ca^2+^ in a molar ratio of 1:1 reduced negamycin migration (spot-shift), implying complex formation (Fig. 6A). At 5-fold molar surplus of Ca^2+^ over negamycin, the antibiotic was almost completely retained at the origin. The addition of Mg^2+^ influenced negamycin migration to a similar extent, while NaCl at a molar surplus of 1:10 showed no effect (Fig. 6A). The ability of negamycin to form a complex with calcium could be confirmed by isothermal titration calorimetry (ITC). Here, binding of Ca^2+^ to negamycin was clearly detected (Fig. 6B), but the affinity of the interaction was low. Due to the low affinity of binding (low c-value titration), ITC did not allow us to determine the stoichiometry of the complex. When manually setting the stoichiometry to 1:1, a binding affinity (K_d_) of 7.98 mM was obtained in Tris buffer pH 7.0 (Fig. 6C). Although the stoichiometry of negamycin and calcium could not be determined with certainty, the K_d_ is not largely dependent on this parameter. When imposing a stoichiometry of 0.5:1 or 2:1, K_d_ values of 8.62 mM or 7.36 mM were obtained, respectively. Furthermore, an effect of the pH on the binding affinity of negamycin and calcium was observed. At pH 8.5, a slightly improved K_d_ of 4.1 mM was determined when fixing the stoichiometry to 1:1. The charges of the different groups (carboxyl, amine) within the negamycin molecule vary with pH as their protonation changes (Fig. S4), which may explain why binding affinities change with pH. Of note, we were not able to detect binding of Mg^2+^ to negamycin by ITC. Most likely, the large heat of dilution resulting from the titration of MgCl_2_ into buffer containing negamycin masked the small quantity of heat released by the binding.

### pH has a substantial impact on negamycin activity.

Negamycin activity was also strongly influenced by the pH of the medium. The pH of 0.5% PP is around 7, and the medium is not buffered. By adjusting the pH of PP to pH 5, an 8-fold reduction in negamycin activity was observed for E. coli, while on the other hand, the MIC improved 2- to 4-fold at pH 8.5 (Fig. 4B). The beneficial effect of calcium on negamycin activity was additive to the alkaline pH, leading to a MIC of 1 μg/ml in E. coli under these optimized conditions (pH 8.5 and 0.5 mM CaCl_2_). Of note, since a CaCl_2_ concentration of 2.5 mM, which had shown the strongest effect on negamycin MIC at pH 7, could not be tested at pH 8.5 due to precipitation, the concentration of CaCl_2_ was lowered to 0.5 mM. Improvement of negamycin activity at pH 8.5/0.5 mM CaCl_2_ was not limited to E. coli but also affected P. aeruginosa and S. aureus, where the negamycin MIC dropped to 4 μg/ml (Fig. 4A).

The pH of the surrounding medium affects the membrane potential of bacteria (32). The electrical potential Δψ across the cytoplasmic membrane of E. coli is more negative at alkaline pH than at acidic pH. The passage of positively charged negamycin molecules across the cytoplasmic membrane may be facilitated by the trans-negative (i.e., surplus negative charge inside) electrical potential gradient. With increasing Δψ at alkaline pH, more negamycin molecules may pass the cytoplasmic membrane in a given time period than at neutral pH, leading to an increased accumulation of negamycin inside the cell. Such behavior has also been reported for the positively charged aminoglycosides (33). However, for the negamycin structure, an additional pH-dependent aspect should be considered. The beta-amino group of negamycin has a pK_a_ of 8.0, meaning that at pH 8.5, the number of negamycin molecules that are zwitterionic is considerably higher than that at pH 7.0 (Fig. S4). At pH 7.0, approximately 99% of negamycin molecules contain a net charge of +1, while only 1% of negamycin is present in its zwitterionic form. At pH 8.5, the two different zwitterionic species of negamycin together make up about 30% of the total amount of molecules, and this net neutral state may contribute to passage across the cytoplasmic membrane, putatively by passive diffusion.

In this regard, we also determined the negamycin MIC of isogenic Δ*acrA*, Δ*acrB*, and Δ*acrAB* mutants under our media conditions. We did not detect a decrease in negamycin MIC in these mutants devoid of the main E. coli efflux pump, neither at pH 7 nor at pH 8.5 plus 0.5 mM CaCl_2_ (Table S2). In contrast, novobiocin MICs were affected heavily by the deletion of this efflux pump, also under alkaline conditions in the presence of CaCl_2_.

By performing experiments with tritium-labeled negamycin, we measured a significantly increased uptake of negamycin at pH 8.5 than at pH 7 (Fig. 4C). At alkaline pH, the amount of accumulated [^3^H]negamycin was increased by 46% and 53% after 15 min and 60 min, respectively. The uptake data directly explain the improved negamycin activity observed at pH 8.5. However, with our experimental setup, we could not detect an increased [^3^H]negamycin accumulation over 60 min when adding CaCl_2_ to the cultures, neither at pH 7 nor at pH 8.5 (Fig. 4C). Similar to the situation at neutral pH, we did not observe a dissipation of the membrane potential or disruption of membrane integrity upon negamycin treatment at pH 8.5, neither in the absence nor presence of CaCl_2_ (Fig. S2 and S3).

### Negamycin susceptibility is reduced in E. coli energy mutants.

**FIG 7:**
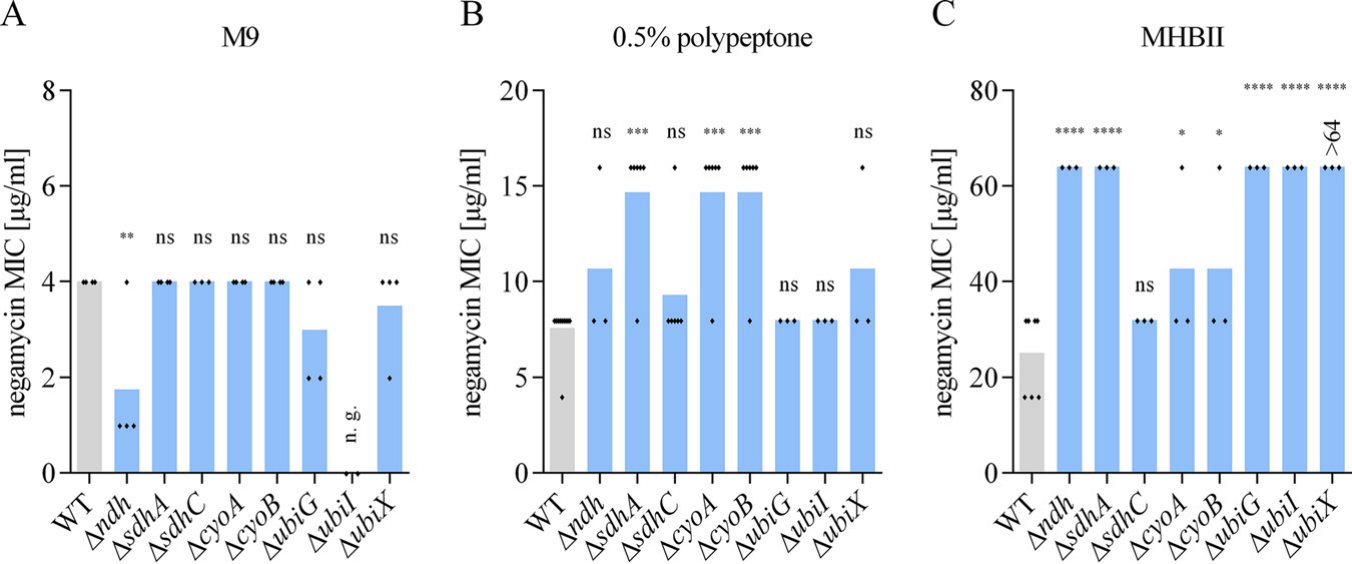
Mutations affecting the respiratory chain reduce negamycin sensitivity in peptide-rich media. Negamycin MICs of different energy mutants of E. coli BW25113 in M9 (A), 0.5% PP (B), or MHBII (C). Each diamond represents an independent MIC determination. Statistical significance was determined using unpaired Student’s *t* test with Holm-Bonferroni correction, comparing the various deletion strains to the wild type (WT). ns, *P* > 0.05; *, *P* ≤ 0.05; **, *P* ≤ 0.01; ***, *P* ≤ 0.001; ****, *P* ≤ 0.0001; n. g., no growth under this condition.

The membrane potential Δψ is dependent on the activity of the respiratory chain complexes (32). Thus, we analyzed several strains of the E. coli Keio collection lacking proteins involved in different stages of the respiratory chain for their susceptibility to negamycin under our media conditions (Fig. 7). In M9 medium, all of the energy mutants tested remained fully susceptible (Fig. 7A). The glucose level in M9 seems sufficient to ensure activity of the ATP-fueled Dpp also in energy mutants, and this transporter is the preferred uptake route in M9. In polypeptone, we observed a 2-fold negamycin MIC increase in some of the mutants (Fig. 7B). Overall, the effects of energy mutations on negamycin susceptibility detected by us were lower than previously reported. Versicor had noted a 2- to 4-fold MIC increase for a negamycin derivative in Δ*hemB*, Δ*ubiD*, and Δ*cydAB* mutants, and more recently, McKinney and coworkers reported an over-4-fold MIC increase of an *ubiX* mutant (24, 25). As we did not observe such substantial effects of the energy mutants under our assay conditions (using M9 or PP), we repeated the MIC determination in MHBII, the medium that had been used before (25). For MHBII, we confirm more pronounced changes with up to 4-fold MIC increase for several of the mutants (Δ*ndh*, Δ*sdhA*, Δ*ubiG*, Δ*ubiI*, Δ*ubiX*) (Fig. 7C). One obvious difference between the three media is their peptide content (0 g/liter in M9, 5 g/liter in 0.5% polypeptone, 17.5 g/liter casein hydrolysate, plus 2 g/liter beef infusion in MHBII). Peptide transporter-based entry routes are less available for negamycin the higher the competition with nutrient peptides, with a stronger impact on Dpp than on DtpD, which puts more emphasis on alternative uptake mechanisms that are more dependent on a certain membrane potential threshold. In addition, it cannot be excluded that metabolic changes in E. coli while growing in the different media play a role.

### Negamycin activity is reduced under anaerobic conditions in peptide-rich media.

**FIG 8:**
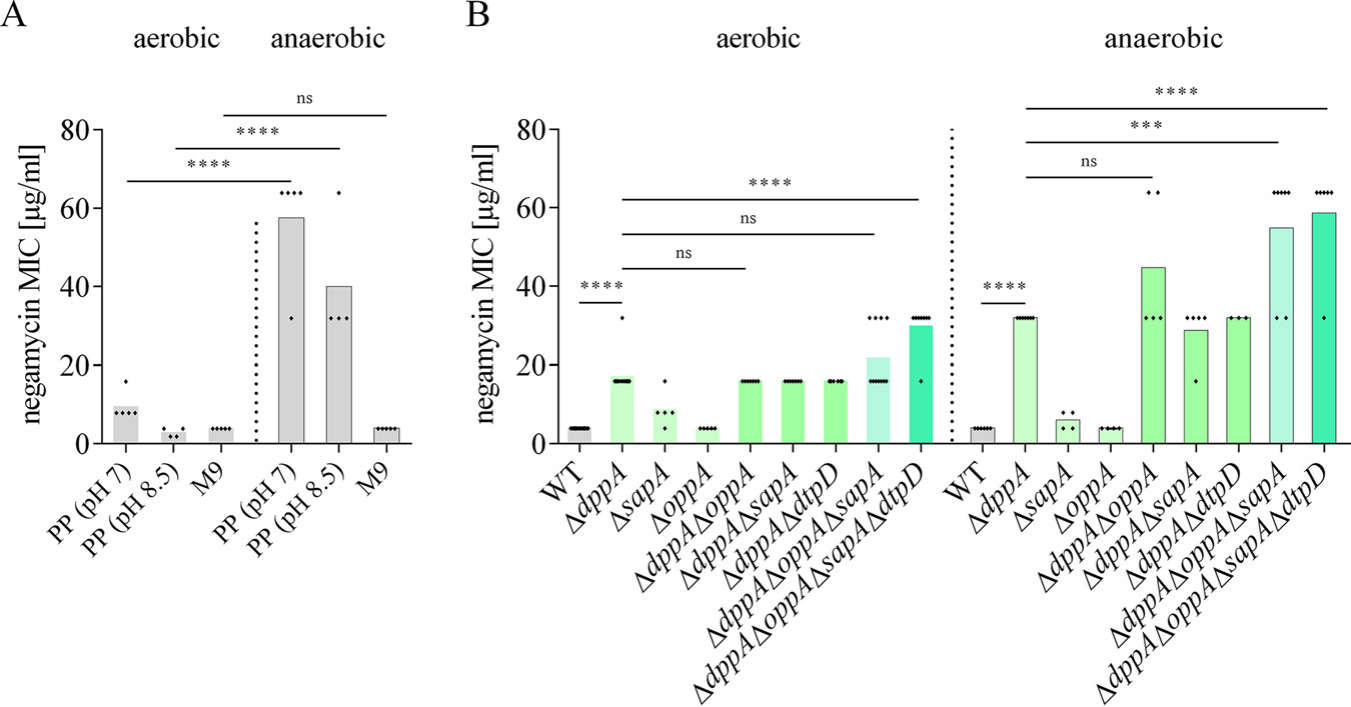
Anaerobic growth conditions reduce negamycin susceptibility in peptide-containing media and in peptide transporter mutants. (A) Negamycin MICs of E. coli BW25113 wild type in different media under aerobic and anaerobic conditions. Statistical significance was determined using Student’s *t* test comparing anaerobic to aerobic growth conditions. (B) Effect of peptide transporter deletions in the E. coli BW25113 background on negamycin susceptibility under aerobic *versus* anaerobic growth conditions in M9 medium. Each diamond represents an independent MIC determination. Statistical significance was determined using unpaired Student's *t* test with Holm-Bonferroni correction. ns, *P* > 0.05; *, *P* ≤ 0.05; ***, *P* ≤ 0.001; ****, *P* ≤ 0.0001.

We also investigated negamycin activity under anaerobic conditions. E. coli BW25113, when growing in PP in the absence of oxygen, was substantially less susceptible to negamycin, and the MIC increased from 8 μg/ml to 32 to 64 μg/ml (Fig. 8A). The same trend was observed for kanamycin and gentamicin in PP (Fig. S5), and it is well described that aminoglycoside activity is strongly impaired under anaerobic conditions (34). Vice versa, when performing the MIC determination in ambient air while shaking the microplate, negamycin MIC was improved by 2-fold in PP most likely as a consequence of the increased oxygen availability (Fig. S6). On the other hand, and in contrast to that of aminoglycosides, the negamycin MIC remained unchanged in M9 minimal medium when incubating the cells under anaerobic conditions (Fig. 8A and Fig. S5). Similar to the results obtained for the energy mutants in M9, the unaltered negamycin MIC probably reflects the availability of Dpp and other peptide transporters for uptake in M9 also under anaerobic conditions. When comparing the set of our single and multiple peptide transporter mutants in M9 under aerobic versus anaerobic conditions, we observed that peptide transporters have a higher impact on negamycin uptake in the latter case (Fig. 8B). Here, the contribution of the two further periplasmic binding proteins OppA and SapA, as well as of the POT DtpD, to negamycin uptake became clearly visible (Fig. 8B). Anaerobiosis also raised the MIC of the quadruple peptide transporter knockout, indicating that at least one further negamycin entry route exists and that this route is oxygen-dependent. An explanation would be the membrane potential-dependent uptake in an aminoglycoside-like manner.

## DISCUSSION

The initial publication on the discovery of negamycin had already described a media dependency of its antibacterial activity (7), providing a first indication that bacterial uptake of this compound may be influenced by the environment. On a molecular level, we now found that the dipeptide-like antibiotic has multiple options to cross the cytoplasmic membrane. In the absence of peptides, negamycin preferably enters the cell by active transport via the dipeptide permease Dpp. Versicor had previously reported a 4-fold MIC increase of a fluorinated negamycin analog in a *dpp* mutant in M9, and the same observation was also made later for unmodified negamycin by AstraZeneca (24, 25). We confirm this primary uptake route by uptake assays using tritium-labeled negamycin (Fig. 2D) as well as by the exclusive selection of Dpp mutants at 2× MIC in M9. Dpp is one of the main E. coli peptide transporters and preferably transports dipeptides composed of l-amino acids along with some tripeptides (27, 35, 36). Its periplasmic binding protein DppA was further shown to be involved in the uptake of 5-aminolevulinic acid as well as heme, proving that Dpp can transport substrates that do not contain a peptide bond (37, 38). It has been postulated that modification of the peptide bond atoms in peptide analogs may lower their binding affinities to DppA (39), a potential explanation for our observation that negamycin uptake is outcompeted at DppA by nutrient peptides.

It is clear that Dpp is not the only uptake route used by negamycin in M9, as only a maximal 4-fold decrease in susceptibility was detected in *dpp* mutants. We found a second periplasmic binding protein, SapA, to be involved in negamycin uptake. Among the seven paralogs of DppA in E. coli, SapA shares the highest homology with DppA and was the only paralog that showed decreased negamycin activity in a single-knockout mutant. The Sap ABC transporter was described to be involved in antimicrobial peptide (AMP) resistance in Salmonella enterica serotype Typhimurium and Haemophilus influenzae (40, 41). In E. coli, the function of the SapA protein in E. coli is unclear. Even though Sap from E. coli shares high amino acid identity with its homolog in *Salmonella*, a function in AMP resistance could not be detected in E. coli. Furthermore, SapBCDF, but not SapA, was shown to contribute to the export of the polyamine putrescine (42). As negamycin activity was decreased, when *sapA* was deleted but not the other subunits of the Sap transporter, SapA may interact with the permease domains of another transporter to support negamycin uptake. Cross talk between periplasmic binding proteins and noncognate permease domains has been described before, e.g., MppA interacts with Dpp for heme transport (38) as well as with Opp for recycling of the cell wall murein (43). Due to the redundancy of transport systems, contributions of single transporters may only become visible when preferred routes are not available. While no decrease in negamycin susceptibility was visible in Δ*oppA* to Δ*oppE* single and Δ*dppA*Δ*oppA* double knockouts in M9, a Δ*dppA*Δ*oppA*Δ*sapA* triple mutant showed a small increase in negamycin MIC compared to that of Δ*dppA*. The contribution of OppA was more clearly visible under anaerobic conditions (Fig. 8B), demonstrating that OppA also plays a minor role in negamycin uptake.

Besides the contribution of different ABC transporters, a decreased susceptibility was also identified in a *dtpD* (*ybgH*) mutant, representing one of the four E. coli proton-dependent oligopeptide transporters (POTs). POTs are found in all kingdoms of life except archaea, and they transport mainly di- and tripeptides but are remarkably promiscuous concerning their substrate specificity (44). Interestingly, POTs play an important role in the absorption of several peptide-like drugs, i.e., the human POT homologs PEPT1 and PEPT2 present in the mammalian brush border and renal membranes transport orally administered beta-lactam antibiotics (45, 46). POTs are less well described for the uptake of antimicrobials by bacteria, but it has been shown in competition assays that E. coli DtpA (YdgR) has a similar substrate specificity as PEPT1, interacting with the aminocephalosporins cefadroxil, cephalexin, and cephradine (47). It has also been suggested recently that uptake of the peptidyl nucleoside blasticidin S could be mediated by POT transporters, especially DtpA and DtpD (48). The presence of a peptide bond is not a prerequisite for substrate recognition by human PEPT1 (49), and the minimal features were described to be a positively charged amino terminus and a negatively charged carboxy terminus separated by at least 4 methyl groups (50), further substantiating that negamycin may serve as a substrate for the E. coli homolog DtpD. Due to redundancy in substrate specificities among the four POTs of E. coli, it cannot be excluded that other POTs besides DtpD contribute to negamycin uptake.

Illicit transport was described for several antimicrobial compounds with peptide-like structural elements, among them pacidamycin and kasugamycin (51, 52). GE81112, a natural tetrapeptide antibiotic, which inhibits protein biosynthesis, was shown to enter the cell via Opp (53). The compound shows very good MICs in minimal media. However, its activity is completely abolished in rich media containing peptides (E. coli MG1655, MIC >512 μg/ml in MHB), and E. coli strains lacking Opp were described to be resistant to GE81112 (53). Similarly, bialaphos, which we used as a comparator for negamycin in this study, is mainly transported via Opp and, to a lesser extent, via Dpp. The deletion of *oppA* increased the bialaphos MIC by 1,000-fold, and a Δ*dppA*Δ*oppA* double mutant showed complete resistance in M9. Comparison with GE81112 and bialaphos emphasizes the particular strength of negamycin in promiscuous uptake. Negamycin is active in minimal salt as well as peptide-containing media, and a quadruple knockout mutant (Δ*dppA*Δ*oppA*Δ*sapA*Δ*dtpD*) still has only an 8-fold reduced susceptibility. This diversity of options is directly reflected by the mutation rates. While negamycin mutation frequencies were high at 2× MIC, they immediately dropped to low rates at 4× MIC. Notably, the risk of spontaneous target mutations is also extremely low. All contacts of negamycin to its target site are established to rRNA (14, 15), and most bacterial species harbor multiple rRNA gene alleles (e.g., 7 rRNA gene copies in E. coli).

Negamycin even showed good activity in PP medium, i.e., when competing with natural peptide substrates at the peptide transporters. Impressively, negamycin can still use peptide transporters under these highly competitive conditions, as indicated by our observation that increasing the amount of polypeptone from 0.5% to 2% raised the negamycin MIC by 4-fold. This observation leads to the question of whether peptide transporters can play a role in negamycin uptake *in vivo*. McKinney and colleagues reported that Dpp was not relevant for negamycin efficacy in a mouse thigh infection (25); nonetheless, the total dose that they required for an equivalent reduction of the bacterial load was increased by 2-fold for the Δ*dppA* mutant compared to that for the wild type. In our MIC assays, we could still detect effects of peptide transporter deletions when peptides were present (e.g., Δ*dtpD* and Δ*sapA*). Thus, although it is clear that negamycin is much less dependent on a single specific peptide transporter than agents that solely rely on illicit transport by a particular route, it still might use such transporters for uptake *in vivo*. However, negamycin also certainly has alternatives to illicit uptake, and the specific environment at the site of infection determines its preferred uptake route.

In peptide-containing media, we detected a substantial impact of calcium on negamycin activity (Table 3), which was particularly beneficial in the concentration range of human blood calcium levels (54, 55). Substituting calcium with magnesium was not effective, but importantly the latter ion also did not negatively affect negamycin activity. In contrast to those of negamycin, aminoglycoside MICs rise in the presence of divalent cations (33). A decreased uptake of streptomycin following the addition of MgCl_2_ and CaCl_2_ was reported for E. coli and P. aeruginosa but also for the Gram-positive S. aureus (56). Divalent cations also hampered the intracellular accumulation of quinolones in E. coli and S. aureus (57–60), and magnesium ions inhibited the uptake of tetracycline in E. coli (61). When testing ciprofloxacin, tetracycline, and gentamicin against E. coli under our experimental conditions, we observed the same trends (Table S1). On the other hand, enhancing effects of calcium on bioactivity have been shown for daptomycin (62). In this case, the substitution of calcium with other divalent cations like Mg^2+^, Ni^2+^, or Mn^2+^ increased daptomycin MICs by at least 32-fold (63). Mechanism-wise, it was suggested that Ca^2+^ is more effective than Mg^2+^ in bridging daptomycin and negatively charged phospholipid headgroups (64), based on the finding that Ca^2+^ seems to fit better between phospholipid headgroups and to interact more strongly with phosphate and carbonyl groups than Mg^2+^ (65). These observations match our data obtained by SAW experiments, where we observed a substantially better binding of negamycin to phospholipid membranes in the presence of Ca^2+^ than of Mg^2+^ (Fig. 5), although a direct interaction of negamycin in solution with both calcium and magnesium was detected by TLC (Fig. 6A). We obtained K_d_ values of 7.98 mM or 4.1 mM by low c-value titration via ITC for calcium binding to negamycin at pH 7 or pH 8.5, respectively. Previously determined binding affinities of other antibiotics to divalent cations were in a similar range, i.e., the quinolone ofloxacin bound Mg^2+^ with a K_d_ of 1 mM as determined by nuclear magnetic resonance (NMR) and tetracycline showed a pH-dependent binding affinity to Ca^2+^ with K_d_ values of 1.1 mM and 0.59 mM at pH 6.5 and pH 7.5, respectively (60, 66). We were not able to determine the stoichiometry of the complex of negamycin and calcium in this study. Tetracycline was shown to bind calcium and magnesium with distinct stoichiometries, namely, one Ca^2+^ or 0.5 Mg^2+^ per tetracycline molecule (66). Since the negamycin-ribosome cocrystal structure revealed a magnesium-mediated contact of negamycin via its carboxylic moiety to the 16S rRNA (14), complex formation of negamycin with Ca^2+^ or Mg^2+^ in solution may involve the carboxyl and also putatively the carbonyl and amine groups. Complexation of quinolones with Mg^2+^ frequently involves both the ketone and deprotonated carboxylate groups (60), while they more rarely bind the metal ion via the two carboxyl oxygen atoms (67). Taken together, calcium significantly improves negamycin activity in Gram-negative and Gram-positive bacteria, in contrast to other antibiotics with intracellular targets (aminoglycosides, fluoroquinolones, tetracyclines), which were shown to be antagonized by divalent cations (Table S1). Enhanced binding to phospholipid membranes mediated by calcium may suggest a role of this cation in negamycin uptake. However, an effect of CaCl_2_ on cell entry did not emerge in our uptake studies with [^3^H]negamycin over a time course of 60 min (Fig. 4C). As calcium binding modifies the physicochemical behavior of negamycin, we cannot exclude an altered distribution behavior of this compound during sample processing in our radioactive assay setup. In addition, we cannot rule out that the beneficial effect of calcium comes into play at a later point in time, which would be difficult to dissect as mixed effects are expected due to the miscoding mode of action of negamycin. On the other hand, our data clearly show that the interaction of negamycin with bacterial membranes stimulated by calcium does not enhance membrane permeability or disturb the membrane potential as a secondary, translation-independent mode of action (Fig. S2 and S3). Thus, the specific molecular mechanisms on how calcium improves negamycin activity needs further investigation.

Our results of decreased negamycin activity at acidic pH, in energy mutants, and under anaerobic conditions indicate that at least one uptake route is affected by the membrane potential. The negative impact of acidic pH on negamycin uptake had previously been noted for E. coli and attributed to the correspondingly lower membrane potential, a characteristic also known for aminoglycosides (25, 68). However, our studies with different growth media emphasize that the impact of the membrane potential on negamycin activity is dependent on the environment. The lack of effect of respiratory chain mutations (Fig. 7A) and oxygen depletion (Fig. 8A) in M9 medium underline that negamycin has entry options that do not rely on a certain membrane potential threshold, such as Dpp. A significant increase in negamycin MICs in different energy mutants was only observed in peptide-containing media (Fig. 7B and C), and the effects were more pronounced in MHBII than in PP. The higher peptide content of MHBII compared to that of PP seems to render peptide transporter routes less accessible and the membrane potential more crucial. Although it has been known for decades that aminoglycoside uptake is membrane potential-dependent, the specific molecular mechanism of translocation of these highly cationic, hydrophilic compounds across the cytoplasmic membrane is not fully elucidated. It remains unclear if yet unidentified transporters or carriers are involved. For negamycin, we have identified a proton-dependent oligopeptide transporter to contribute to uptake. The main driving force of this secondary active transporter family is the membrane potential, as these transporters couple substrate translocation to proton flux down an electrochemical proton gradient (47).

With regard to increased uptake at alkaline pH (Fig. 4C), a second factor deserves consideration besides the membrane potential, namely, microspeciation of negamycin. The number of net neutral negamycin molecules increases with pH, reaching about 30% at pH 8.5 (Fig. S4). Fluoroquinolones and tetracyclines have been proposed to diffuse passively through the lipid bilayer in their uncharged forms (69). At physiological pH, zwitterionic ciprofloxacin is predominant over the neutral (uncharged) form. It was proposed that zwitterionic molecules form stacks, which reduces their polarity and favors insertion into the bilayer. Neutralization is achieved by the intramolecular transfer of protons favored by partial solvation loss, and ciprofloxacin crosses the membrane by passive diffusion as a neutral monomer (70). Whether a zwitterionic fraction of negamycin might also be able to cross the cytoplasmic membrane without an active transport process remains speculative considering that negamycin is notably more hydrophilic than ciprofloxacin.

In summary, our data show that negamycin is exceptional in its capacity to employ multiple, independent uptake routes across the bacterial cytoplasmic membrane, which highlights the very promiscuous nature of this small natural product, thereby decreasing the risk of high-level resistance by transporter mutations.

## MATERIALS AND METHODS

### Bacterial strains and growth conditions.

Bacterial strains used in this study are listed in Table S3. Strains were grown in either 0.5% polypeptone (PP) in water (BD BBL polypeptone, catalog no. 211910), M9 minimal medium (47.74 mM Na_2_HPO_4_ × 2H_2_O, 22.04 mM KH_2_PO_4_, 8.56 mM NaCl, 18.7 mM NH_4_Cl, 2 mM MgSO_4_, 100 μM CaCl_2_, 0.4% glucose), lysogeny broth (LB), Mueller-Hinton broth (MHB; BD Difco), or cation-adjusted Mueller-Hinton broth (MHBII; BD Difco) at 37°C with shaking (190 rpm) or on respective agar. Antibiotics, salts, or PP were added as indicated. Negamycin (>95% purity) was synthesized by Squarix GmbH, Marl, Germany, based on published procedures (20, 71). Tritium-labeling of negamycin was performed by Hartmann Analytic GmbH, Braunschweig, Germany. Bialaphos was obtained from Alfa Aesar, tetracycline from Sigma-Aldrich Chemie GmbH, streptomycin sulfate from AppliChem, and gentamicin sulfate and kanamycin sulfate from Carl Roth. The pH was adjusted to the indicated values using 1 M HCl or 1 M NaOH. For growth curve experiments, strains were cultivated in microplates with shaking at intervals, and the optical density at 600 nm (OD_600_) was recorded in a microplate reader (Infinite M200 PRO, Tecan).

### Determination of antibacterial activity.

The MIC was determined by the broth microdilution method according to the Clinical and Laboratory Standards Institute (CLSI) guidelines using the direct colony suspension method with an inoculum of 5 × 10^5^ CFU/ml (72). In addition to the standard MHB, antimicrobial susceptibility testing was performed using different media and conditions. MICs were read after incubation at 37°C for 20 h or after 24 h when M9 minimal medium was used. Anaerobic growth conditions were generated using the GasPAK Anaerobe Pouch System (BD), and the incubation time in M9 medium was extended to 28 h under anaerobic conditions. All values were determined in at least three independent experiments, i.e., different bacterial cultures grown on different days were subjected to the same experimental procedures. Each independent measurement is represented as a diamond in the dot plot overlaid on the bar chart, and the arithmetic mean of the single measurements is displayed as a colored, vertical bar (Fig. 2, 4, 7, and 8). Statistical analysis was performed using R (the R Foundation for Statistical Computing, version 3.6.1). Unpaired Student’s *t* test was used for statistical evaluation. For multiple comparisons, the Holm-Bonferroni correction was applied. A *P* value ≤0.05 was considered statistically significant.

### *In vitro* transcription-translation assay.

An *in vitro* coupled transcription/translation assay was performed to assess the potency of negamycin on bacterial translation in a cell-free system. The assay is based on S30 extracts prepared from logarithmically growing E. coli strain MRE600 (73), an RNase I deficient strain, according to a procedure described by Zubay (74). The system further uses the pBESTluc plasmid (Promega Corporation, Madison, USA) encoding the firefly (Photinus pyralis) luciferase gene under the control of a *tac* promoter as a reporter, allowing the detection of translation inhibition by measuring luminescence. Apart from S30 extract and pBESTluc plasmid, the reaction mixture contained the following supplements: 2.5 mM ATP, 0.5 mM GTP, 0.5 mM UTP, 0.5 mM CTP, 20 amino acids (0.04 mM each), an ATP regenerating system (creatine phosphokinase/phosphocreatine), 3.2% (wt/vol) polyethylene glycol 600, 8 mM putrescine, and 2 mM dithiothreitol (DTT) in an appropriate buffer system (40 mM triethylamine [pH 7.5], 140 mM potassium acetate, 8 mM magnesium acetate, 20 mM ammonium acetate, 1.4 mM spermidine). *In vitro* coupled transcription-translation reactions in the presence of a concentration series of negamycin were performed for 2 h at 25°C. After addition of the substrate luciferin, chemiluminescence was recorded in a multiplate reader (Infinite M200, Tecan). The IC_50_ was determined as the concentration of negamycin, which led to 50% reduction of luminescence compared to an untreated control.

### Miscoding assay.

A K31STOP mutation was introduced into the pBESTluc plasmid by site-directed mutagenesis using the primers Luc-mut_for and Luc-mut_rev (Table S4). The resulting plasmid pBESTluc-mut harbors a stop codon in the firefly luciferase gene that leads to a truncated, nonfunctional enzyme. For the miscoding assay, E. coli BW25113 carrying the pBESTluc-mut plasmid was grown to the exponential phase. The culture was diluted to an OD_600_ of 0.1 and treated with either negamycin, streptomycin (positive control), or tetracycline (negative control) at sub-MICs. After incubation in M9 for 5 h at 37°C, OD_600_ and chemiluminescence were measured following the addition of 2 mM luciferin in a microplate reader (Infinite M200 PRO, Tecan). Miscoding (readthrough) activity was detected as the ratio of relative light units (RLU) per OD_600_.

### Knockout of genes in E. coli.

Knockout strains were constructed as previously described by Datsenko and Wanner (75). Briefly, the pKD3 (chloramphenicol) or pKD13 (kanamycin) resistance cassette was amplified using primers with homologous ends to the gene of interest. The used primers are listed in Table S4. E. coli cells were first transformed with the helper plasmid pKD46 and then with the extended resistance cassette. Recombination took place during incubation for 24 h at 37°C. The resistance cassette was subsequently deleted using the FLP-expression plasmid pCP20. Gene deletions were confirmed by PCR followed by Sanger sequencing. For the deletion of multiple genes in the same strain, the single-gene knockout strains of the Keio collection (76) were used as the templates.

### Complementation of *dppA* in E. coli.

The *dppA* gene was amplified by PCR from genomic DNA of E. coli BW25113 using the primers dppA-XbaI_for and dppA-XhoI_rev (Table S4) and cloned into pASK-IBA5 (IBA GmbH, Göttingen). The resulting plasmid pASK-*dppA* was used for E. coli strain JW3513 (Δ*dppA*) transformation. Expression of *dppA* in M9 medium was induced by adding 10 ng/ml anhydrotetracycline (Cayman Chemical, USA).

### [^3^H]negamycin uptake.

Radiolabeled [^3^H]negamycin with a specific activity of 16.6 Ci/mmol was acquired and used in all uptake assays after dilution with unlabeled compound to a final specific activity of 0.052 Ci/mmol. E. coli BW25113 or its isogenic Δ*dppA* mutant were grown in M9 or PP under shaking conditions at 37°C until early exponential growth phase. Cells were harvested by centrifugation at room temperature and resuspended in fresh medium to an OD_600_ of 5. The cell suspension was shaken at 37°C for 10 min before 32 μg/ml of [^3^H]negamycin (specific activity: 0.052 Ci/mmol) were added and cells were further incubated at 37°C under shaking conditions. 500 μl samples were taken after 15 min and 60 min and quickly cooled on ice. Samples were centrifuged, washed two times with 500 μl ice-cold 3% NaCl (wt/vol), and transferred into a precooled tube containing 800 μl silicone oil (two parts of AR 200 [Sigma-Aldrich] to one part of AK 100 [Wacker]). After centrifugation, the aqueous phase and the silicone oil were removed. The cell pellet was resuspended in 100 μl ice-cold 3% NaCl (wt/vol) and transferred to a liquid scintillation counting vial. 1 ml Soluene (PerkinElmer) was added and incubated over night at room temperature. The next day, 3 ml of the liquid scintillation cocktail Ultima Gold (PerkinElmer) was added, mixed thoroughly, and incubated for 3 h at room temperature. Samples were measured (counts per minute [cpm] over 10 min) using the liquid scintillation analyzer Tri-Carb 2900TR (Packard Bioscience) with the software package QuantaSmart 1.31 (Packard Bioscience). Negamycin amounts (μg) were calculated based on a dilution series of the [^3^H]negamycin compound standard (specific activity: 0.052 Ci/mmol) and put in relation to the E. coli cell number, determined by OD_600_, which was measured in parallel when samples were taken. Statistical significance was calculated using R (the R Foundation for Statistical Computing, version 3.6.1) as described in the methods for determination of antimicrobial activity.

### Isolation of spontaneous antibiotic-resistant mutants.

A single E. coli BW25113 colony was transferred in 10 ml LB and incubated overnight at 37°C with shaking (190 rpm). The overnight culture was centrifuged and resuspended in 0.9% saline (0.9% wt/vol of NaCl in MilliQ water), and 1× 10^8^ to 5 × 10^8^ CFU were plated on M9 or PP agar, containing different concentrations of either negamycin or bialaphos. Agar plates were incubated at 37°C, and colonies grown after 24 h were transferred onto a fresh agar plate containing either negamycin or bialaphos and checked for growth. In cases where the resistance rate turned out to be high, the experiment was repeated with lower cell numbers per plate to allow for precise colony counting.

### Sequencing of the *dpp* operon of negamycin resistant mutants.

The *dpp* operon was amplified from genomic DNA of the negamycin resistant mutants selected on either M9 or 0.5% PP media using the primers dppA-F-2 and dppA-F rev2 (Table S4). The resulting PCR products (size approximately 8.5 kB) were subjected to Sanger sequencing (LGC Genomics, Germany) using the primers listed in Table S4. Since no PCR product was obtained for several of the negamycin resistant mutants selected on M9, one of these mutants (M9-2) was subjected to whole-genome sequencing. After detection of a large chromosomal deletion in M9-2, the same deletion was confirmed by PCR and Sanger sequencing in 8 other mutants using the primers dpp-flank_for and dpp-flank_rev (Table S4).

### Whole-genome sequencing.

Genomic DNA from overnight cultures of E. coli strains BW25113 wild type and M9-2, a negamycin resistant mutant selected on M9 agar at 2× MIC which had not yielded a PCR product for the *dpp* operon (dppA-F-2 and dppA-F rev2 primers) was purified using the MasterPure Gram-positive DNA purification kit (Epicentre Biotechnologies). Shotgun libraries with an insert size of approximately 300 bp of the different E. coli strains were generated by fragmentation followed by end repair of DNA (Eurofins Genomics GmbH). The libraries were sequenced on Illumina MiSeq using chemistry v3, and the obtained reads were mapped on the reference genome of E. coli strain K-12 substrain MG1655 (NCBI accession number NC_000913.3) (Eurofins Genomics GmbH).

### SAW biosensor measurements.

Surface acoustic wave (SAW) measurements were performed to investigate the membrane-binding capacity of negamycin using a sam®5 blue sensor device (SAW Instruments GmbH, Bonn, Germany) as described before (77). Binding events were measured by means of phase shifts of the acoustic wave. SAW sensor chips were cleaned as described, followed by preincubation with a chloroform solution of 10 mM hexadecanethiol overnight. After drying the quartz, a model membrane consisting of either POPC or POPC with 10 mol% DOPG was formed by Langmuir-Blodgett technique and transferred to the sensors as described before (78). After embedding the dried sensor into the device and rehydrating the model membrane with ultrapure water, binding of negamycin to the membrane was investigated in a 200 mM morpholinepropanesulfonic acid (MOPS) (pH 7) buffer flow after injecting negamycin in a dilution series ranging from 7.5 × 10^−8^ M to 1 × 10^−5^ M.

### Thin-layer chromatography.

Thin-layer chromatography (TLC) was performed on TLC silica gel 60 F254 (Merck) with chloroform-methanol-25% aqueous ammonia (2:2:1) as mobile phase. 6 μl of each sample containing negamycin in a concentration of 6 mM without or with the respective salts (CaCl_2_, MgCl_2_, or NaCl) in molar ratios of 1:1, 1:5, or 1:10 were applied to the adsorbent layer. The developed plates were dried at 60°C, treated with ninhydrin for visualization, and dried for another 15 min at 100°C.

### Isothermal titration calorimetry (ITC).

Binding of negamycin to Ca^2+^ was determined by isothermal titration calorimetry (ITC). Experiments were performed by titrating 50 mM CaCl_2_ into the cell filled with 0.5 mM negamycin. Both components were dissolved in the same buffer (200 mM Tris, pH 7 or 8.5) to prevent unspecific heat caused by dilution effects. Subsequently, the first point of the isotherm and signals from three different control experiments (titration of 50 mM CaCl_2_ into buffer, buffer into 0.5 mM negamycin, and buffer into buffer) were subtracted from the isotherm before data evaluation. All ITC experiments were performed on a Microcal PEAQ-ITC (Malvern) at 25°C with a stirring speed of 750 rpm and 2 consecutive experiments of 19 injections of 2 μl each. The two data sets of 19 injections each were combined using the Malvern MicroCal Concat tool. The resulting isotherm was integrated and fitted with the one side binding model of the corresponding Malvern MicroCal PEAQ-ITC analysis software.

### Cell membrane integrity assay.

The impact of antibiotic treatment on cell membrane integrity was investigated using the cell-impermeant nucleic acid dye SYTOX Green (Invitrogen). E. coli BW25113 was grown in PP pH 7 or pH 8.5 to the exponential growth phase, and cultures were diluted to an OD_600_ of 0.05 in fresh medium without or with CaCl_2_ (2.5 mM at pH 7 and 0.5 mM at pH 8.5). SYTOX Green was added to the cells to a final concentration of 5 μM, and the cell suspensions were incubated for 5 min at room temperature. Samples of 100 μl were transferred to a black 96-well microplate and antibiotics were added at the indicated concentrations. Immediately after antibiotic addition, fluorescence emission at 510 to 650 nm was measured with a wavelength step size of 2 nm after excitation at 475 nm in a microplate reader (Spark, Tecan). Fluorescence emission spectra were recorded every 10 min over a time course of 3 h at 30°C, with periodic shaking before each measurement. Of note, the SYTOX Green dye showed an increased fluorescence intensity in PP pH 8.5 compared to that in PP pH 7 even in the absence of cells, correlating with the higher relative fluorescence units (RFU) measured in the untreated control samples at pH 8.5 compared to those at pH 7.

### Membrane potential assay.

The membrane potential upon antibiotic treatment was assessed using the DiOC_2_(3) dye (Molecular Probes, Fisher Scientific). To minimize dye efflux, the assay was performed using E. coli BW25113 Δ*acrA*. E. coli BW25113 Δ*acrA* was grown in PP pH 7 or pH 8.5 to the exponential growth phase, and the OD_600_ was adjusted to 0.5 in fresh medium with or without CaCl_2_ (2.5 mM at pH 7 and 0.5 mM at pH 8.5). 30 μM DiOC_2_(3) was added and samples were incubated at room temperature for 15 min in the dark. Volumes of 100 μl were transferred to a black 96-well microplate and the red/green fluorescence (excitation at 485 nm, emission at 530 nm [green] and 630 nm [red]) was measured in a microplate reader (Infinite M200 PRO, Tecan). After an initial fluorescence measurement, the program was paused and antibiotics were added at the indicated concentrations. Thereafter, the red/green fluorescence was recorded every 5 min over a time course of 3 h at 25°C.

## Supplementary Material

Supplemental file 1
