# Supplementary material for: The Antibiotic Negamycin Crosses the Bacterial Cytoplasmic Membrane by Multiple Routes
Source: Antimicrob Agents Chemother. 2021 Mar 18;65(4):e00986-20. doi: 10.1128/AAC.00986-20 (PMC8097410; doi:10.1128/AAC.00986-20)
Supplement: Supplemental file 1 [file AAC.00986-20-s0002.pdf]

Supplemental Data

The antibiotic negamycin crosses the bacterial cytoplasmic membrane by multiple routes

Daniel Hörömpöli[a,b]+, Catherine Ciglia[c]+, Karl-Heinz Glüsenkamp[d], Lars Ole Haustedt[e], Hildegard Falkenstein-Paul[f], Gerd Bendas[f], Anne Berscheid[a,b,c]*#, Heike Brötz-Oesterhelt[a,b,c,g]*#

[a] Interfaculty Institute of Microbiology and Infection Medicine, Department of Microbial Bioactive Compounds, University of Tuebingen, Tuebingen, Germany

[b] German Center of Infection Research (DZIF), partner site Tuebingen

[c] Institute of Pharmaceutical Biology, University of Duesseldorf, Duesseldorf, Germany

[d] Squarix GmbH, Marl, Germany

[e] AnalytiCon Discovery GmbH, Potsdam, Germany

[f] Pharmaceutical Institute, Department of Pharmaceutical & Cell Biological Chemistry, University of Bonn, Bonn, Germany

[g] Cluster of Excellence 2124: Controlling Microbes to Fight Infection

Running Head: Negamycin passage across the cytoplasmic membrane

+ D.H. and C.C. contributed equally to this work. Author order was determined by mutual verbal consent.

* H.B.-O. and A.B. share senior authorship.

# Address correspondence to Heike Brötz-Oesterhelt, heike.broetz-oesterhelt@uni-tuebingen.de; Anne Berscheid, anne.berscheid@uni-tuebingen.de

Figures

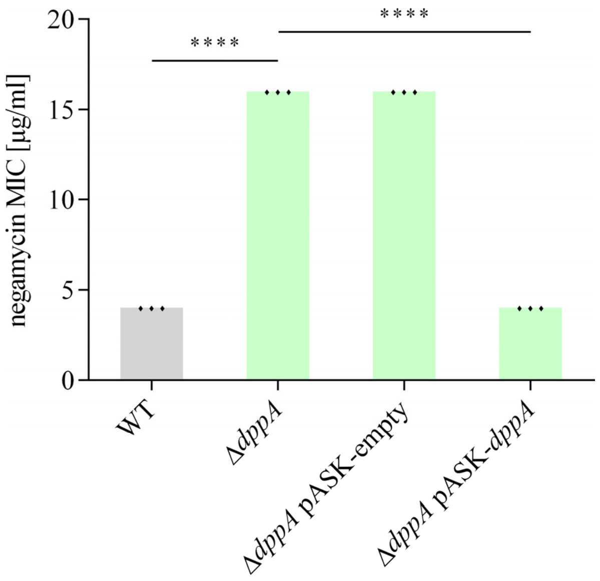

Figure S1. Deletion of chromosomal *dppA* in *E. coli* BW25113 reduces negamycin susceptibility and can be complemented by DppA expression from a plasmid (pASK-*dppA*). The same plasmid lacking *dppA* (pASK-empty) served as a negative control. Negamycin MICs were determined in M9. DppA expression was induced with 10 ng/ml anhydrotetracycline. Each diamond represents an independent MIC determination. Statistical significance was determined using unpaired Student's t-test with Holm-Bonferroni correction. ****, $P \leq 0.0001$.

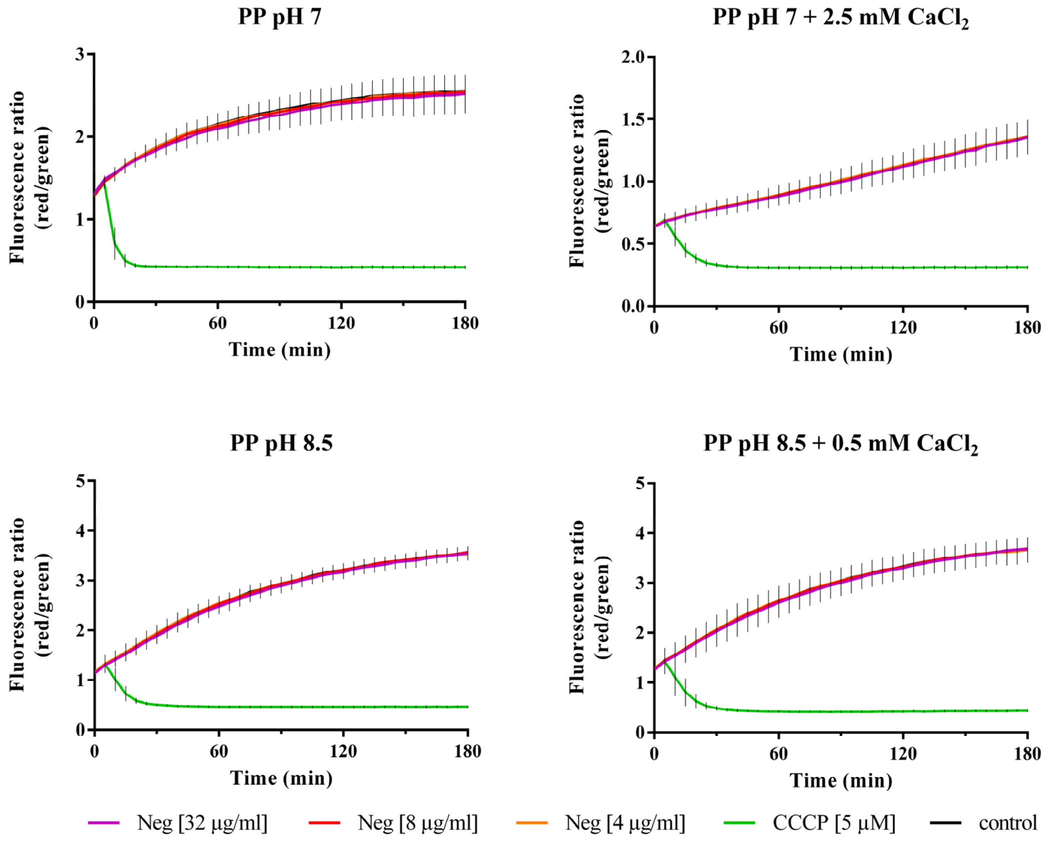

Figure S2. Negamycin treatment does not lead to rapid membrane depolarization under different media conditions. *E. coli* BW25113 Δ*acrA* was used for the $DiOC_2(3)$ dye-based membrane potential assay to minimize dye efflux. Negamycin (Neg) was applied at different concentrations up to 32 µg/ml (corresponding to 4x MIC at pH 7 and to 32-64x MIC at pH 8.5 + 0.5 mM $CaCl_2$). No dissipation of the membrane potential was observed over a time course of 180 min in 0.5% polypeptone medium (PP) at pH 7 and pH 8.5, neither in the absence nor in the presence of $CaCl_2$, while the addition of the protonophore CCCP led to a quick depolarization of the *E. coli* cells indicated by the decrease in the red/green fluorescence ratio under all media conditions. The experiment was performed in at least three biological replicates, graphs represent the mean ± standard deviation.

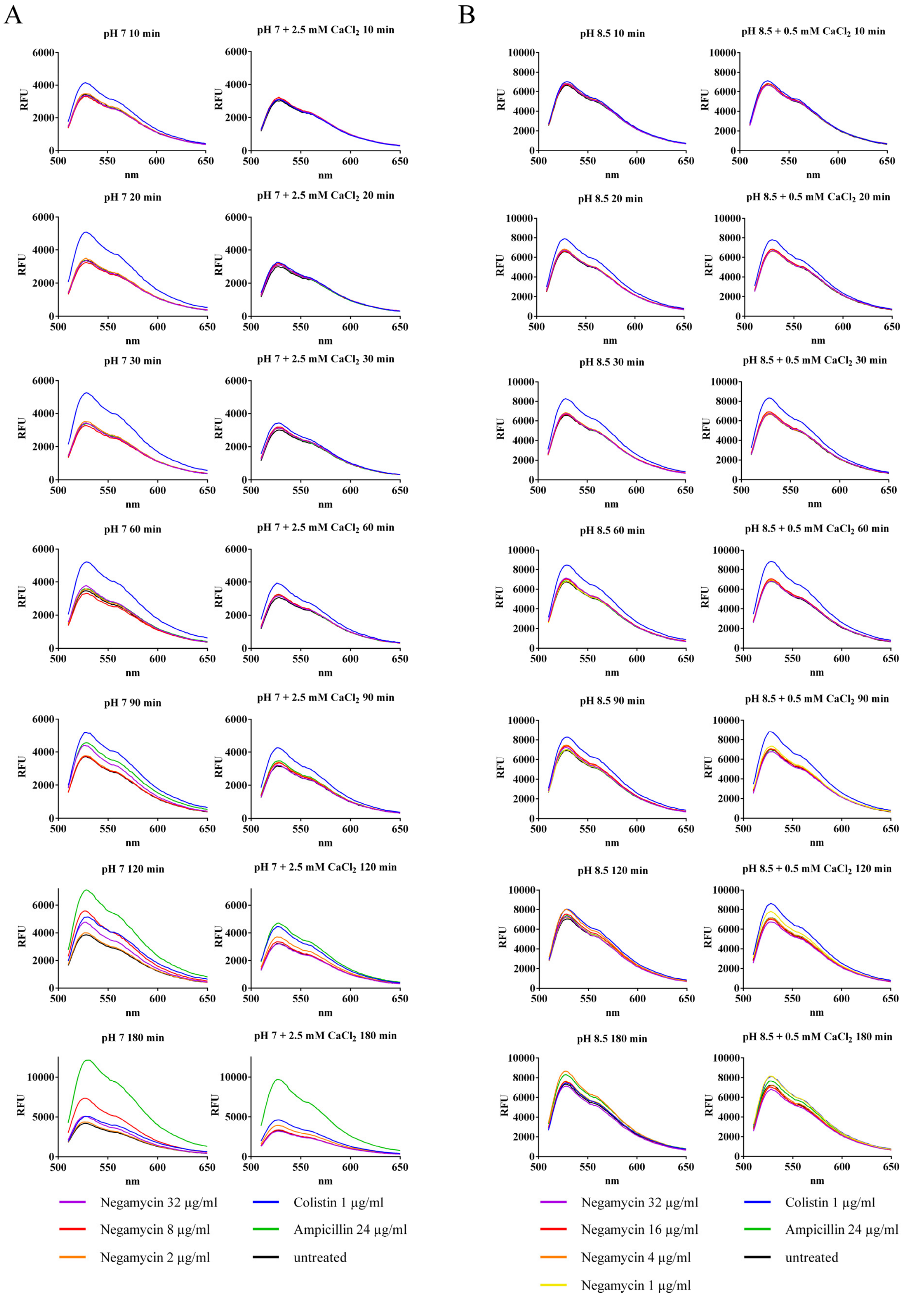
A
pH 7 10 min
pH 7 + 2.5 mM CaCl2 10 min
pH 7 20 min
pH 7 + 2.5 mM CaCl2 20 min
pH 7 30 min
pH 7 + 2.5 mM CaCl2 30 min
pH 7 60 min
pH 7 + 2.5 mM CaCl2 60 min
pH 7 90 min
pH 7 + 2.5 mM CaCl2 90 min
pH 7 120 min
pH 7 + 2.5 mM CaCl2 120 min
pH 7 180 min
pH 7 + 2.5 mM CaCl2 180 min
RFU
nm
Negamycin 32 µg/ml
Negamycin 8 µg/ml
Negamycin 2 µg/ml
Colistin 1 µg/ml
Ampicillin 24 µg/ml
untreated
B
pH 8.5 10 min
pH 8.5 + 0.5 mM CaCl2 10 min
pH 8.5 20 min
pH 8.5 + 0.5 mM CaCl2 20 min
pH 8.5 30 min
pH 8.5 + 0.5 mM CaCl2 30 min
pH 8.5 60 min
pH 8.5 + 0.5 mM CaCl2 60 min
pH 8.5 90 min
pH 8.5 + 0.5 mM CaCl2 90 min
pH 8.5 120 min
pH 8.5 + 0.5 mM CaCl2 120 min
pH 8.5 180 min
pH 8.5 + 0.5 mM CaCl2 180 min
RFU
nm
Negamycin 32 µg/ml
Negamycin 16 µg/ml
Negamycin 4 µg/ml
Negamycin 1 µg/ml
Colistin 1 µg/ml
Ampicillin 24 µg/ml
untreated

Figure S3. Negamycin treatment does not lead to a rapid permeabilization of the cytoplasmic membrane in the presence of $CaCl_2$. Membrane integrity upon antibiotic treatment was assessed using the cell membrane impermeant nucleic acid stain SYTOX Green, that emits increased fluorescence when binding to nucleic acids after having gained access through membrane pores. Negamycin concentrations up to 32 µg/ml (i.e. corresponding to 4x MIC in PP pH 7 and 16x MIC at pH 7 + 2.5 mM $CaCl_2$) did not lead to an increased permeability of *E. coli* BW25113 within 60 min of treatment, neither at pH 7 ± $CaCl_2$ (A) nor at pH 8.5 ± $CaCl_2$ (B). After 90 min of negamycin treatment, first effects on membrane integrity appeared, which, however, can be most likely attributed to the miscoding activity of this compound. The effect was best visible at concentrations reflecting the MIC in the respective media (i.e. 8 µg/ml at pH 7, 2 µg/ml in pH 7 + 2.5 mM $CaCl_2$, 4 µg/ml at pH 8.5 and 1 µg/ml at pH 8.5 + 0.5 mM $CaCl_2$), in agreement with the fact that negamycin, although having miscoding as the primary mechanism, leads to full translation inhibition at higher concentrations. Similarly, aberrant membrane proteins resulting from translational miscoding were also suggested to be responsible for membrane permeabilization during prolonged aminoglycoside exposure (1, 2). Importantly, $CaCl_2$ addition did not accelerate the permeabilization process, also not under alkaline conditions. In contrast, the membrane disrupting agent colistin disturbed the membrane integrity already after 10-20 min at 4x MIC, as indicated by the increase in fluorescence signal compared to the untreated control. Cell lysis caused by the treatment with the cell wall inhibitor ampicillin became visible after 90-120 min of treatment in accordance with the fact that peptidoglycan sacculus weakening requires some time to take effect. The graph shows the data representative of three independent biological experiments. Of note, SYTOX Green shows a higher fluorescence intensity at alkaline pH, even in the absence of cells.

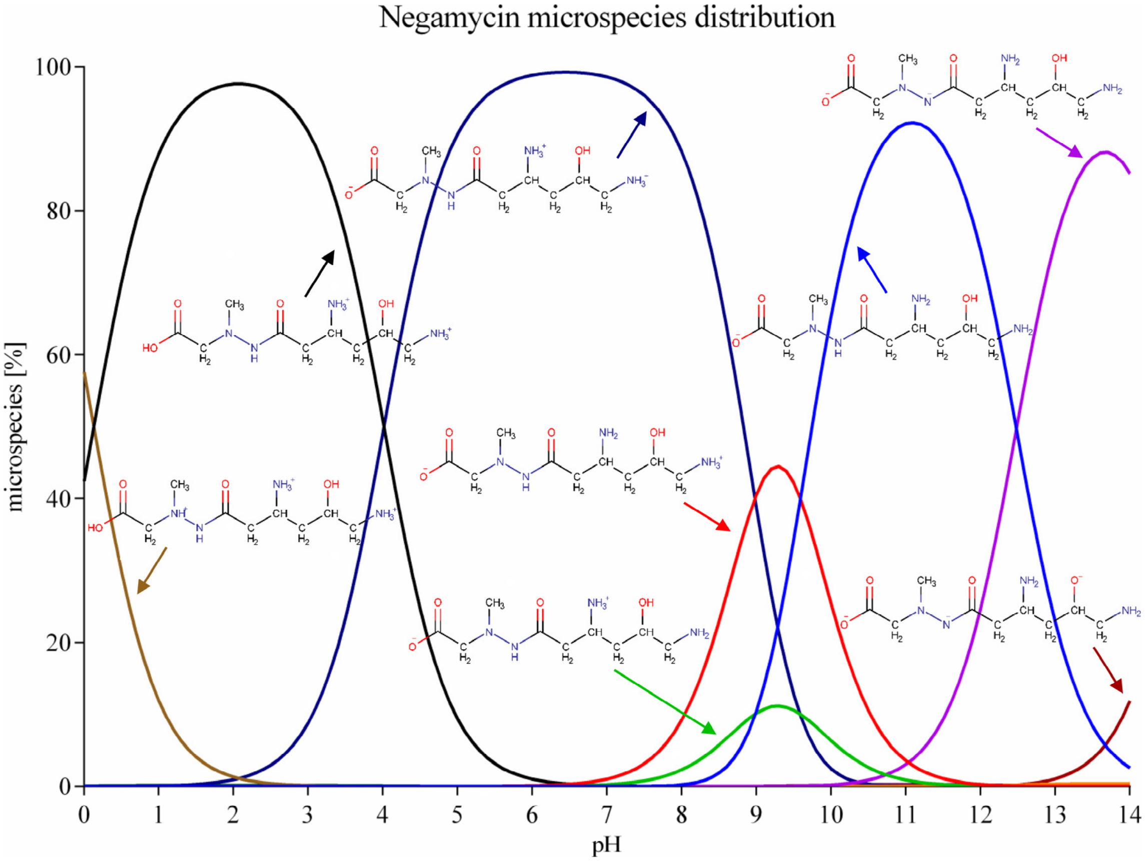

Figure S4. Negamycin microspecies distribution calculated with ChemAxon MarvinView (version 17.29.0). Calculated pKa values (4.02, 8.88 and 9.69) by ChemAxon differ slightly from the experimentally determined pKa values (3.2, 8.0 and 9.6) by Guo et al. (3).

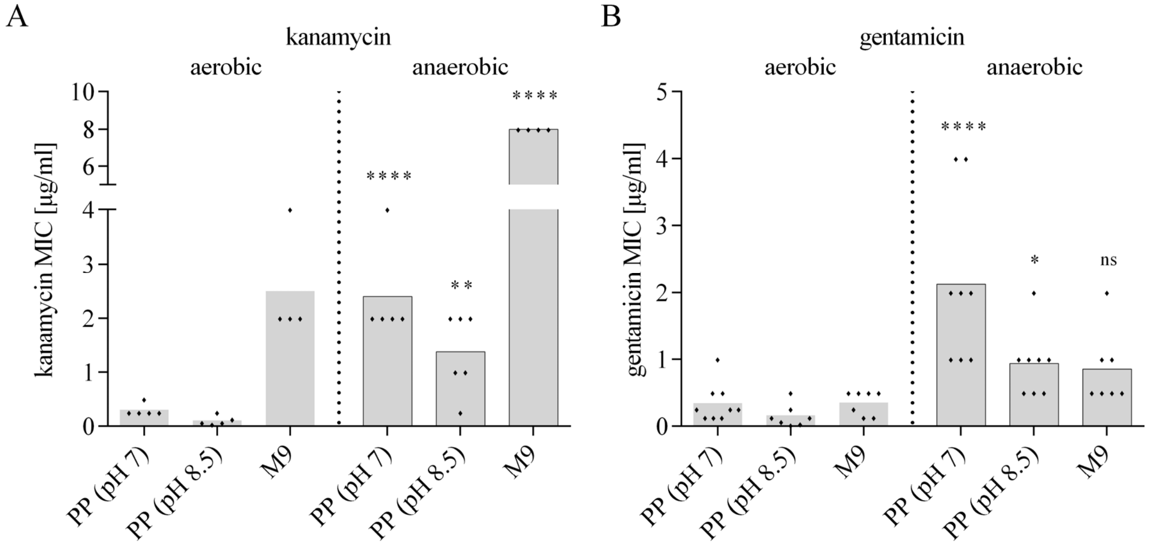

Figure S5. Susceptibility of *E. coli* BW25113 to the aminoglycosides kanamycin (A) and gentamicin (B) under aerobic and anaerobic growth conditions in 0.5% polypeptone (PP) pH 7 or pH 8.5 or in M9 minimal medium. Each diamond represents an independent MIC determination. Statistical significance was determined using unpaired Student's t-test comparing the effect of anaerobic growth conditions to the same antibiotic within the same medium under aerobic growth conditions. ns, $P > 0.05$; *, $P \leq 0.05$; **, $P \leq 0.01$; ***, $P \leq 0.001$; ****, $P \leq 0.0001$.

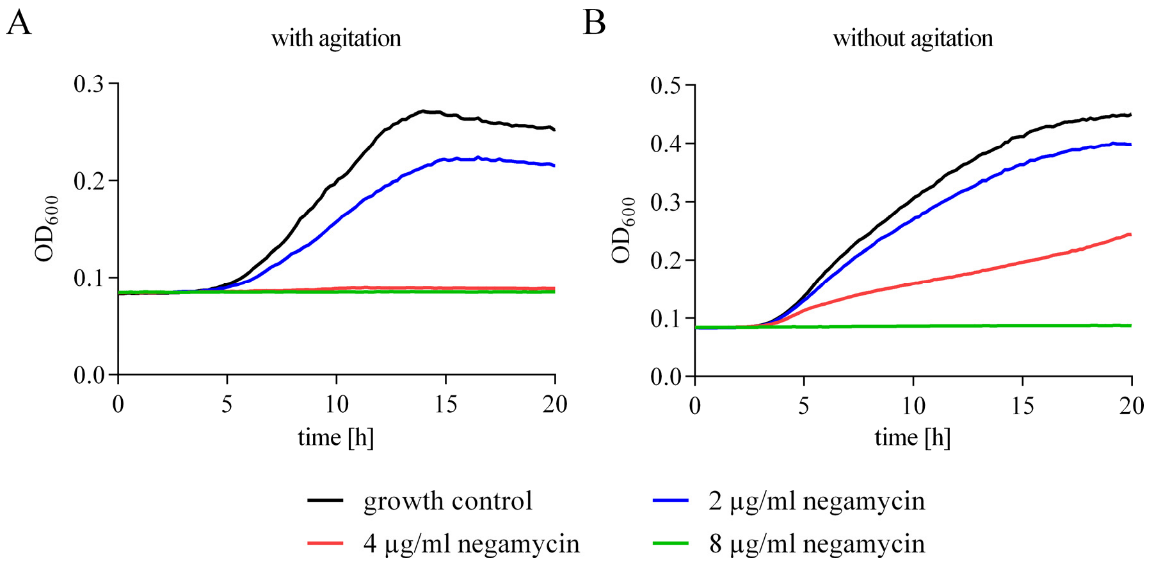

Figure S6. Effect of negamycin on the growth of *E. coli* BW25113 in 0.5% polypeptone (PP) in microplates with (A) or without (B) agitation. Microplates were either incubated with periodic shaking (218 rpm for 30 s at 5 min intervals) (A) or kept static as customary during MIC determinations (B) and OD_{600} was measured in a microplate reader (Infinite M200 pro, TECAN). Negamycin susceptibility is increased under well-aerated growth conditions in PP.

Tables

Table S1. Antimicrobial activity of ciprofloxacin, tetracycline and gentamicin against *E. coli* BW25113 in the presence of different concentrations of salts added to 0.5% polypeptone medium.

	MIC [µg/ml]									
		$CaCl_2$			$MgCl_2$			NaCl		
	0 mM	2.5 mM	10 mM	50 mM	2.5 mM	10 mM	50 mM	2.5 mM	10 mM	50 mM
Ciprofloxacin	0.008	0.008	0.016	0.06	0.008	0.016	0.125	0.004	0.004	0.008
Tetracycline	2	4-8	4	16	8	16	>64	2	2	1-2
Gentamicin	0.06	0.06	0.06	1	0.06	0.06	0.25	0.06	0.25	0.5

Table S2. Antibacterial activity of negamycin and novobiocin in efflux deficient mutants in PP pH 7 and PP pH 8.5 with 0.5 mM $CaCl_2$. Deletion of the main *E. coli* efflux pump AcrAB did not improve negamycin activity under either growth condition, while the activity of the known AcrAB substrate novobiocin strongly increased in the pump deletion mutant.

	MIC [µg/ml]							
	Negamycin				Novobiocin			
	WT	Δ*acrA*	Δ*acrB*	Δ*acrAB*	WT	Δ*acrA*	Δ*acrB*	Δ*acrAB*
pH 7	8	8	8	16	32	1-2	1-2	0.5
pH 8.5 + 0.5 mM $CaCl_2$	0.5-1	0.5	0.5	1	>64	32	32	4-8

Table S3. Bacterial strains used in this study.

Strain	Gene function (4)	Genotype/characteristics	source
E. coli **ATCC 25922™**		Clinical isolate, CLSI reference strain	(5)
E. coli **BW25113**		wildtype; F-, Δ(*araD-araB*)*567*, Δ*lacZ4787*(::*rrnB*-3), λ-, *rph*-1, Δ(*rhaD-rhaB*)*568*, *hsdR514*	(6, 7)
E. coli **JW0422 Δ*cyoA***	cytochrome bo_3 ubiquinol oxidase subunit II	F-, Δ*(araD-araB*)*567*, Δ*lacZ4787*(::*rrnB*-3), λ-, Δ*cyoA789::kan*, *rph*-1, Δ(*rhaD-rhaB*)*568*, *hsdR514*	(6, 7)
E. coli **JW0421 Δ*cyoB***	cytochrome bo_3 ubiquinol oxidase subunit II	F-, Δ*(araD-araB*)*567*, Δ*lacZ4787*(::*rrnB*-3), λ-, Δ*cyoB788::kan*, *rph*-1, Δ(*rhaD-rhaB*)*568*, *hsdR514*	(6, 7)
E. coli **JW0711 Δ*sdhC***	succinate:quinone oxireductase	F-, Δ*(araD-araB*)*567*, Δ*lacZ4787*(::*rrnB*-3), λ-, Δ*sdhC771::kan*, *rph*-1, Δ(*rhaD-rhaB*)*568*, *hsdR514*	(6, 7)
E. coli **JW0713 Δ*sdhA***	succinate:quinone oxireductase, FAD binding protein	F-, Δ*(araD-araB*)*567*, Δ*lacZ4787*(::*rrnB*-3), λ-, Δ*sdhA773::kan*, *rph*-1, Δ(*rhaD-rhaB*)*568*, *hsdR514*	(6, 7)
E. coli **JW1095 Δ*ndh***	NADH:quinone oxireductase II	F-, Δ*(araD-araB*)*567*, Δ*lacZ4787*(::*rrnB*-3), λ-, Δ*ndh771::kan*, *rph*-1, Δ(*rhaD-rhaB*)*568*, *hsdR514*	(6, 7)
E. coli **JW2226 Δ*ubiG***	3-demethylubiquinone-*8* 3-*O*-methyltransferase, 2-octaprenyl-6-hydroxyphenol methylase	F-, Δ*(araD-araB*)*567*, Δ*lacZ4787*(::*rrnB*-3), λ-, Δ*ubiG785::kan*, *rph*-1, Δ(*rhaD-rhaB*)*568*, *hsdR514*	(6, 7)
E. coli **JW2874 Δ*ubiI***	2-octaprenylphenol hydroxylase	F-, Δ*(araD-araB*)*567*, Δ*lacZ4787*(::*rrnB*-3), λ-, Δ*ubiI757::kan*, *rph*-1, Δ(*rhaD-rhaB*)*568*, *hsdR514*	(6, 7)
E. coli **JW2308 Δ*ubiX***	flavin prenyltransferase	F-, Δ*(araD-araB*)*567*, Δ*lacZ4787*(::*rrnB*-3), λ-, Δ*ubiX732::kan*, *rph*-1, Δ(*rhaD-rhaB*)*568*, *hsdR514*	(6, 7)
E. coli **JW3513 Δ*dppA***	dipeptide ABC transporter periplasmic binding protein	F-, Δ(*araD-araB*)*567*, Δ*lacZ4787*(::*rrnB*-3), λ-, Δ*dppA728::kan*, *rph*-1, Δ(*rhaD-rhaB*)*568*, *hsdR514*	(6, 7)
E. coli **JW3513 Δ*dppA* pASK-*dppA***	dipeptide ABC transporter periplasmic binding protein	F-, Δ(*araD-araB*)*567*, Δ*lacZ4787*(::*rrnB*-3), λ-, Δ*dppA728*,*rph*-1, Δ(*rhaD-rhaB*)*568*, *hsdR514*, pASK-*dppA*	this study
E. coli **JW3513 Δ*dppA* pASK-empty**	dipeptide ABC transporter periplasmic binding protein	F-, Δ(*araD-araB*)*567*, Δ*lacZ4787*(::*rrnB*-3), λ-, Δ*dppA728*,*rph*-1, Δ(*rhaD-rhaB*)*568*, *hsdR514*, pASK-empty	this study
E. coli **JW3512 Δ*dppB***	dipeptide ABC transporter membrane subunit	F-, Δ(*araD-araB*)*567*, Δ*lacZ4787*(::*rrnB*-3), λ-, Δ*dppB726::kan*, *rph*-1, Δ(*rhaD-rhaB*)*568*, *hsdR514*	(6, 7)
E. coli **JW3511 Δ*dppC***	dipeptide ABC transporter membrane subunit	F-, Δ(*araD-araB*)*567*, Δ*lacZ4787*(::*rrnB*-3), λ-, Δ*dppC725::kan*, *rph*-1, Δ(*rhaD-rhaB*)*568*, *hsdR514*	(6, 7)
E. coli **JW3510 Δ*dppD***	dipeptide ABC transporter ATP binding subunit	F-, Δ(*araD-araB*)*567*, Δ*lacZ4787*(::*rrnB*-3), λ-, Δ*dppD724::kan*, *rph*-1, Δ(*rhaD-rhaB*)*568*, *hsdR514*	(6, 7)

E. coli* JW3509 Δ*dppF	dipeptide ABC transporter ATP binding subunit	F-, Δ(*araD-araB*)*567*, Δ*lacZ4787*(::*rrnB*-3), *λ*-, Δ*dppF723::kan*, *rph*-1, Δ(*rhaD-rhaB*)*568*, *hsdR514*	(6, 7)
E. coli* JW1287 Δ*sapA	dipeptide ABC transporter periplasmic binding protein	F-, Δ(*araD-araB*)*567*, Δ*lacZ4787*(::*rrnB*-3), *λ*-, Δ*sapA730::kan*, *rph*-1, Δ(*rhaD-rhaB*)*568*, *hsdR514*	(6, 7)
E. coli* JW1286 Δ*sapB	peptide ABC transporter membrane subunit	F-, Δ*(araD-araB)567*, Δ*lacZ4787*(::*rrnB*-3), $λ^-$, *ΔsapB729::kan*, *rph*-1, Δ*(rhaD-rhaB)568*, *hsdR514*	(6, 7)
E. coli* JW1285 Δ*sapC	dipeptide ABC transporter membrane subunit	F-, Δ(*araD-araB*)*567*, Δ*lacZ4787*(::*rrnB*-3), *λ*-, Δ*sapC728::kan*, *rph*-1, Δ(*rhaD-rhaB*)*568*, *hsdR514*	(6, 7)
E. coli* JW1284 Δ*sapD	dipeptide ABC transporter ATP binding subunit	F-, Δ(*araD-araB*)*567*, Δ*lacZ4787*(::*rrnB*-3), *λ*-, Δ*sapD727::kan*, *rph*-1, Δ(*rhaD-rhaB*)*568*, *hsdR514*	(6, 7)
E. coli* JW1283 Δ*sapF	dipeptide ABC transporter ATP binding subunit	F-, Δ(*araD-araB*)*567*, Δ*lacZ4787*(::*rrnB*-3), *λ*-, Δ*sapF726::kan*, *rph*-1, Δ(*rhaD-rhaB*)*568*, *hsdR514*	(6, 7)
E. coli* JW1235 Δ*oppA	oligopeptide ABC transporter periplasmic binding protein	F-, Δ(*araD-araB*)*567*, Δ*lacZ4787*(::*rrnB*-3), *λ*-, Δ*oppA750::kan*, *rph*-1, Δ(*rhaD-rhaB*)*568*, *hsdR514*	(6, 7)
E. coli* JW1236 Δ*oppB	oligopeptide ABC transporter membrane subunit	F-, Δ(*araD-araB*)*567*, Δ*lacZ4787*(::*rrnB*-3), *λ*-, Δ*oppB751::kan*, *rph*-1, Δ(*rhaD-rhaB*)*568*, *hsdR514*	(6, 7)
E. coli* JW1237 Δ*oppC	oligopeptide ABC transporter membrane subunit	F-, Δ(*araD-araB*)*567*, Δ*lacZ4787*(::*rrnB*-3), *λ*-, Δ*oppC752::kan*, *rph*-1, Δ(*rhaD-rhaB*)*568*, *hsdR514*	(6, 7)
E. coli* JW1238 Δ*oppD	oligopeptide ABC transporter ATP binding subunit	F-, Δ(*araD-araB*)*567*, Δ*lacZ4787*(::*rrnB*-3), *λ*-, Δ*oppD753::kan*, *rph*-1, Δ(*rhaD-rhaB*)*568*, *hsdR514*	(6, 7)
E. coli* JW1239 Δ*oppF	oligopeptide ABC transporter ATP binding subunit	F-, Δ(*araD-araB*)*567*, Δ*lacZ4787*(::*rrnB*-3), *λ*-, Δ*oppF754::kan*, *rph*-1, Δ(*rhaD-rhaB*)*568*, *hsdR514*	(6, 7)
E. coli* JW1322 Δ*mppA	peptide ABC transporter periplasmic binding protein	F-, Δ(*araD-araB*)*567*, Δ*lacZ4787*(::*rrnB*-3), *λ*-, Δ*mppA767::kan*, *rph*-1, Δ(*rhaD-rhaB*)*568*, *hsdR514*	(6, 7)
E. coli* JW2988 Δ*ygiS	putative deoxycholate binding periplasmic protein	F-, Δ(*araD-araB*)*567*, Δ*lacZ4787*(::*rrnB*-3), *λ*-, Δ*ygiS790::kan*, *rph*-1, Δ(*rhaD-rhaB*)*568*, *hsdR514*	(6, 7)
E. coli* JW5111 *ΔgsiB	glutathione ABC transporter periplasmic binding protein	F-, Δ(*araD-araB*)*567*, Δ*lacZ4787*(::*rrnB*-3), *λ*-, Δ*gsiB730::kan*, *rph*-1, Δ(*rhaD-rhaB*)*568*, *hsdR514*	(6, 7)
E. coli* JW5240 *ΔddpA	putative dipeptide ABC transporter periplasmic binding protein	F-, Δ(*araD-araB*)*567*, Δ*lacZ4787*(::*rrnB*-3), *λ*-, Δ*ddpA780::kan*, *rph*-1, Δ(*rhaD-rhaB*)*568*, *hsdR514*	(6, 7)
E. coli* JW3441 *ΔnikA	nickel ABC transporter periplasmic binding protein	F-, Δ(*araD-araB*)*567*, Δ*lacZ4787*(::*rrnB*-3), *λ*-, Δ*nikA730::kan*, *rph*-1, Δ(*rhaD-rhaB*)*568*, *hsdR514*	(6, 7)
***E. coli* JW0699 Δ*dtpD* (Δ*ybgH*)**	dipeptide:H^+ symporter	F-, Δ(*araD-araB*)*567*, Δ*lacZ4787*(::*rrnB*-3), *λ*-, Δ*ybgH759::kan*, *rph*-1, Δ(*rhaD-rhaB*)*568*, *hsdR514*	(6, 7)
***E. coli* JW1626 Δ*dtpA* (Δ*ydgR*)**	dipeptide/tripeptide:H^+ symporter	F-, Δ(*araD-araB*)*567*, Δ*lacZ4787*(::*rrnB*-3), *λ*-, Δ*ydgR784::kan*, *rph*-1, Δ(*rhaD-rhaB*)*568*, *hsdR514*	(6, 7)

***E. coli* JW4091 Δ*dtpC* (Δ*yjdL*)**	dipeptide/tripeptide:H^+ symporter	F-, Δ(*araD-araB*)*567*, Δ*lacZ4787*(::*rrnB*-3), λ-, Δ*yjdL757::kan*, , *rph*-1, Δ(*rhaD-rhaB*)*568*, *hsdR514*	(6, 7)
***E. coli* JW3463 Δ*dtpB* (Δ*yhiP*)**	dipeptide/tripeptide:H^+ symporter	F-, Δ(*araD-araB*)*567*, Δ*lacZ4787*(::*rrnB*-3), λ-, Δ*yhiP752::kan*, , *rph*-1, Δ(*rhaD-rhaB*)*568*, *hsdR514*	(6, 7)
E. coli* JW2143 Δ*lysP	lysine:H^+ symporter	F-, *Δ(araD-araB)567*, *ΔlacZ4787*(::*rrnB*-3), λ^-, *ΔlysP783::kan*, *rph-1*, *Δ(rhaD-rhaB)568*, *hsdR514*	(6, 7)
E. coli* JW2303 Δ*hisP	histidine/lysine/arginine/ornithine ABC transporter, ATP binding subunit	F-, *Δ(araD-araB)567*, *ΔlacZ4787*(::*rrnB*-3), λ^-, *ΔhisP778::kan*, *rph-1*, *Δ(rhaD-rhaB)568*, *hsdR514*	(6, 7)
E. coli* JW4093 Δ*cadB	lysine:cadaverine antiporter	F-, *Δ(araD-araB)567*, *ΔlacZ4787*(::*rrnB*-3), λ^-, *ΔcadB759::kan*, *rph-1*, *Δ(rhaD-rhaB)568*, *hsdR514*	(6, 7)
E. coli* JW2305 Δ*hisQ	histidine/lysine/arginine/ornithine ABC transporter, membrane subunit	F-, *Δ(araD-araB)567*, *ΔlacZ4787*(::*rrnB*-3), λ^-, *ΔhisQ780::kan*, *rph-1*, *Δ(rhaD-rhaB)568*, *hsdR514*	(6, 7)
E. coli* JW2306 Δ*hisJ	histidine ABC transporter, periplasmic binding protein	F-, *Δ(araD-araB)567*, *ΔlacZ4787*(::*rrnB*-3), λ^-, *ΔhisJ730::kan*, *rph-1*, *Δ(rhaD-rhaB)568*, *hsdR514*	(6, 7)
E. coli* JW2307 Δ*argT	lysine/arginine/ornithine ABC transporter, periplasmic binding protein	F-, *Δ(araD-araB)567*, *ΔlacZ4787*(::*rrnB*-3), λ^-, *ΔargT721::kan*, *rph-1*, *Δ(rhaD-rhaB)568*, *hsdR514*	(6, 7)
E. coli* JW2304 Δ*hisM	histidine/lysine/arginine/ornithine ABC transporter, membrane subunit	F-, *Δ(araD-araB)567*, *ΔlacZ4787*(::*rrnB*-3), λ^-, *ΔhisM779::kan*, *rph-1*, *Δ(rhaD-rhaB)568*, *hsdR514*	(6, 7)
E. coli* Δ*dppA*Δ*sapA	multiple peptide transporters	F-, Δ(*araD-araB*)*567*, Δ*lacZ4787*(::*rrnB*-3), λ-, Δ*dppA728::kan*, Δ*sapA::cm*, *rph*-1, Δ(*rhaD-rhaB*)*568*, *hsdR514*	this study
E. coli* Δ*dppA*Δ*oppA	multiple peptide transporters	F-, Δ(*araD-araB*)*567*, Δ*lacZ4787*(::*rrnB*-3), λ-, Δ*dppA728::kan*, Δ*oppA::cm*, *rph*-1, Δ(*rhaD-rhaB*)*568*, *hsdR514*	this study
E. coli* Δ*dppA*Δ*dtpD	multiple peptide transporters	F-, Δ(*araD-araB*)*567*, Δ*lacZ4787*(::*rrnB*-3), λ-, Δ*dppA728*, Δ*ybgH759::kan*, *rph*-1, Δ(*rhaD-rhaB*)*568*, *hsdR514*	this study
E. coli* Δ*dppA*Δ*sapA*Δ*oppA	multiple peptide transporters	F-, Δ(*araD-araB*)*567*, Δ*lacZ4787*(::*rrnB*-3), λ-, Δ*dppA728*, Δ*sapA730*, Δ*oppA750::kan*, *rph*-1, Δ(*rhaD-rhaB*)*568*, *hsdR514*	this study
E. coli* Δ*dppA*Δ*sapA*Δ*oppA*Δ*dtpD	multiple peptide transporters	F-, Δ(*araD-araB*)*567*, Δ*lacZ4787*(::*rrnB*-3), λ-, Δ*dppA728*, Δ*sapA730*, Δ*oppA750*, Δ*ybgH759::kan*, *rph*-1, Δ(*rhaD-rhaB*)*568*, *hsdR514*	this study
E. coli* Δ*acrA	Multidrug efflux pump membrane protein	F-, *Δ(araD-araB)567*, *ΔlacZ4787*(::*rrnB*-3), λ^-, *ΔacrA7480::kan*, *rph-1*, *Δ(rhaD-rhaB)568*, *hsdR514*	(6, 7)
E. coli* Δ*acrB	Multidrug efflux pump permease	F-, *Δ(araD-araB)567*, *ΔlacZ4787*(::*rrnB*-3), λ^-, *ΔacrB747::kan*, *rph-1*, *Δ(rhaD-rhaB)568*, *hsdR514*	(6, 7)
E. coli* Δ*acrA*Δ*acrB	Multidrug efflux pump	F-, Δ(*araD-araB*)*567*, Δ*lacZ4787*(::*rrnB*-3), λ-, Δ*(acrB747-acrA748)::kan*, *rph*-1, Δ(*rhaD-rhaB*)*568*, *hsdR514*	this study

***Pseudomonas aeruginosa* PAO1**	reference strain	(8, 9)
***Staphylococcus aureus* ATCC 29213**	reference strain	(5)
***Bacillus subtilis* 168 trpC2**	*trpC2*	(10)

Table S4: Oligonucleotides used in this study.

Oligonucleotide	Sequence (5'-3')
**Knockout generation**	
**oppA-H1-pKD3**	AGAGAAGTTTAGTAGCAGCTGGCGTTCTGGCTGCGCTAATGGTGTAGGCTGGAGCTGCTTC
**oppA-H2-pKD3**	CCGGGTATAGGTATTATCCAGCGGATCTTTGCCGGTATAGCCATATGAATATCCTCCTTAG
**sapA-H1-pKD3**	TGAATCTCCCCCGCATGCTGATATCCGCGACAGCGGTTTTGGTGTAGGCTGGAGCTGCTTC
**sapA-H2-pKD3**	CCAGCAAAGGAGGCGTTACCAAACGGGCTAAGTACCAGACCATATGAATATCCTCCTTAG
**dtpD-H1-pKD13**	GCGCAACTATTACGTGACGTTGCCGTCAATTGTGGAATTATTATGTGTAGGCTGGAGCTG
**dtpD-H2-pKD13**	GCATCAGGCATCGGTGCTGGCCTATTAAGACTCCAGCGCCAGCGCATCCGGGGATCCGT
**DppA complementation**	
**dppA-XbaI_for**	TTGGTCTAGATTGGAGCAGAATAATGCGTA
**dppA-XhoI_rev**	TTGCCTCGAGTTGCCTTTGCCATCAGTCTT
**Dpp operon PCR amplification and sequencing**	
**dppA-F-2**	TGACAACTCGGTGACCTATG
**dppA-F-1**	GATTGACGAGGGCGTATCTG
**dppA-F 0**	CCGCCTTATTCGACCTACAC
**dppA-F 1**	CTCACCGTTGGTGTAAATCC
**dppA-F 2**	AAGGCATGGGCTTGCCAGAG
**dppA-F 3**	GCTGAAAGAAGCGGGTCTGG
**dppA-F 4**	CCATCCGGCAGCAATACTTC
**dppA-F 5**	CCGTCGCCCATATGATCTTG
**dppA-F 6**	ATGTGCTGTCGCGCCTGATG
**dppA-F 7**	GCATTTCGGCGACGAAAGCG
**dppA-F 8**	CGCGTCACCCGTATACTCAG
**dppA-F 9**	CGAGCACTATGACCGCTATC
**dppA-F rev**	ATTGTGTCGTGCCTCATTCC
**dppA-F rev2**	GCAGCGATCATTGCTTATCC
**Characterization and sequencing of the 95 kb deletion including the *dpp* operon**	
**dpp-flank_for**	GCAATAGATAACCACGGGAAGG
**dpp-flank_rev**	TTTACGGAAGGTCGTTACCAG
**dpp-flank2_for**	TTATTGCTTATACATGATC
**dpp-flank3_for**	ATGACGGTCAGATATTAT
**Miscoding assay**	
**Luc-mut_for**	ACTGCATAAGGCTATGTAGAGATACGCCCTGGT
**Luc-mut_rev**	ACCAGGGCGTATCTCTACATAGCCTTATGCAGT

References

1. Davis BD, Chen LL, Tai PC. 1986. Misread protein creates membrane channels: an essential step in the bactericidal action of aminoglycosides. Proc Natl Acad Sci U S A 83:6164-8.
2. Busse HJ, Wöstmann C, Bakker EP. 1992. The bactericidal action of streptomycin: membrane permeabilization caused by the insertion of mistranslated proteins into the cytoplasmic membrane of *Escherichia coli* and subsequent caging of the antibiotic inside the cells due to degradation of these proteins. Journal of General Microbiology 138:551-61.
3. Guo J, Miele EW, Chen A, Luzietti RA, Zambrowski M, Walsky RL, Buurman ET. 2015. Pharmacokinetics of the natural antibiotic negamycin. Xenobiotica 45:625-633.
4. Keseler IM, Mackie A, Santos-Zavaleta A, Billington R, Bonavides-Martinez C, Caspi R, Fulcher C, Gama-Castro S, Kothari A, Krummenacker M, Latendresse M, Muniz-Rascado L, Ong Q, Paley S, Peralta-Gil M, Subhraveti P, Velazquez-Ramirez DA, Weaver D, Collado-Vides J, Paulsen I, Karp PD. 2017. The EcoCyc database: reflecting new knowledge about *Escherichia coli* K-12. Nucleic Acids Res 45:D543-D550.
5. Patel JB, Cockerill FR, Bradford PA, Eliopoulos GM, Hindler JA, Jenkins SG, Lewis JS, Limbago B, Miller LA, Nicolau DP, Powell M, Swenson JM, Turnidge JD, Weinstein MP, Zimmer BL. 2015. Methods for dilution antimicrobial susceptibility tests for bacteria that grow aerobically. Approved Standard - Tenth Edition, vol 35. Clinical and Laboratory Standards Institute, USA.
6. Baba T, Ara T, Hasegawa M, Takai Y, Okumura Y, Baba M, Datsenko KA, Tomita M, Wanner BL, Mori H. 2006. Construction of *Escherichia coli* K-12 in-frame, single-gene knockout mutants: the Keio collection. Molecular Systems Biology 2:1-11.
7. Datsenko KA, Wanner BL. 2000. One-step inactivation of chromosomal genes in *Escherichia coli* K-12 using PCR products. Proceedings of the National Academy of Sciences of the United States of America 97:6640-5.
8. Holloway BW. 1955. Genetic recombination in *Pseudomonas aeruginosa*. J Gen Microbiol 13:572-81.
9. Stover C, Pham X, Erwin A, Mizoguchi S, Warrener P, Hickey M, Brinkman F, Hufnagle W, Kowalik D, Lagrou M. 2000. Complete genome sequence of *Pseudomonas aeruginosa* PAO1, an opportunistic pathogen. Nature 406:959-964.
10. Anagnostopoulos C, Spizizen J. 1961. Requirements for Transformation in *Bacillus subtilis*. Journal of Bacteriology 81:741-6.
